# Network Analysis of Predicted Therapeutic Symptoms in National Health Insurance Herbal Prescriptions

**DOI:** 10.3390/life15111769

**Published:** 2025-11-18

**Authors:** Seokwoo Jang, Ahyoug Lee, Changwon Kho

**Affiliations:** 1 School of Korean Medicine, Pusan National University, Yangsan 50612, Republic of Korea; jds7913@pusan.ac.kr (S.J.); ahyoung.lee@pusan.ac.kr (A.L.); 2 Division of Applied Medicine, School of Korean Medicine, Pusan National University, Yangsan 50612, Republic of Korea

**Keywords:** bioinformatics, National Health Insurance Herbal prescriptions, network analysis, Traditional Chinese Medicine, Traditional Korean Medicine

## Abstract

Background: National Health Insurance Herbal prescriptions (NHPs) are widely used; however, their multi-component composition complicates mechanistic interpretation and impedes the development of evidence-based approaches in traditional medicine and healthcare policy. In this study, we applied a systems biology approach to link molecular mechanisms to clinical effects. Methods: From 56 NHPs, 13 with sufficient clinical evidence were selected. Multi-layer networks connecting herbs, ingredients, genes, and diseases were constructed using SymMap, with interactions filtered for oral bioavailability and statistical significance (false discovery rate < 0.05). Network-predicted diseases were validated against a clinically validated benchmark using permutation-based null model analysis, and gene set enrichment analysis (GSEA) was used to identify key molecular pathways. Results: Networks predicted an average of 1359 diseases per NHP, reflecting their polypharmacology. Importantly, the overall predicted disease sets for 10 of 13 NHPs showed statistically significant overlap with known clinical uses (*p* < 0.05, several with *p* < 0.001). GSEA indicated that NHPs commonly modulate three biological axes—hormone–metabolic regulation, neural signaling, and cell proliferation control. Conclusions: NHPs act as potential systemic homeostasis regulators. Our study introduces a computationally validated framework integrating network pharmacology with permutation-based statistical testing, providing a data-driven rationale for NHP use. These computational findings are exploratory and require future biological and clinical validation.

## 1. Introduction

National Health Insurance Herbal Prescriptions (NHPs), which are widely used in Traditional Korean Medicine (TKM), were first introduced in 1987 by the Federation of Korean Medical Insurance Societies (now the Health Insurance Review & Assessment Service). This began with 26 combined-extract formulations and 68 single-extract formulations. The implementation of NHPs has significantly enhanced quality control by standardizing the production processes and requiring pharmaceutical companies to submit their ingredient profiles. This standardization also ensured the accurate identification of the ingredients in herbal formulations.

By 2025, 56 combined-extract formulations and 68 single-extract formulations have been designated as NHPs. These are manufactured by pharmaceutical companies in various forms and supplied to Korean medicine clinics and hospitals. However, elucidating the molecular mechanisms of NHPs and other herbal medicines remains challenging. Each component herb contains multiple compounds rather than a single active ingredient, rendering mechanistic analysis complex. For example, according to the Traditional Chinese Medicine (TCM) database SymMap, *Panax ginseng* contains 84 compounds that can affect hundreds of target genes [1]. This issue has become increasingly urgent amidst a growing global demand for scientifically validated, evidence-based traditional medicine. Furthermore, as NHPs are covered by South Korean National Health Insurance, elucidating their molecular mechanisms is essential to justify their continued policy support and ensure the efficient use of public healthcare resources. Thus, various approaches are required to predict the effects of these multi-compound formulations, among which bioinformatics plays a pivotal role.

Bioinformatics integrates computational and statistical methods to collect, analyze, and interpret large-scale biological data. Notable studies using bioinformatics include those by Baek et al. [2], who identified target organs for the active components of Radix Achyranthis Bidentatae through bioinformatic methods, and Park et al., who used the K-HERB Network database to identify herbal medicines applicable to heart failure through network analysis [3]. Such studies have demonstrated that bioinformatics can be effectively utilized in TKM and TCM to assess the similarities between existing prescriptions and facilitate the development of new ones.

Recent methodological advances in network pharmacology, exemplified by Pan et al.’s CHM-FIEFP framework [4], highlight the importance of multi-level validation integrating computational, experimental, and clinical data. Our study followed this trend by emphasizing robust statistical correction and transparency while positioning our framework primarily as a hypothesis-generating tool pending future experimental validation.

In South Korea, the K-HERB Network has been developed to model the components and effects of various herbal prescriptions in a network format [5]. However, the K-HERB Network has limitations. For herbal formulas, it only references articles from the Oriental Medicine Advanced Searching Integrated System (OASIS), a traditional medicine information portal for prescriptions, and for herbology, it includes information on herbs based solely on domestic Korean medicine articles and those available in PubMed. Moreover, there are insufficient data on the specific compounds in herbs, the target proteins they affect, and the diseases associated with these proteins.

In this study, we utilized TKM databases and bioinformatics techniques to construct molecular-based networks for 13 selected NHPs. We then compared the diseases predicted from these networks against a “gold standard” curated from the clinical literature. To address the limitations of prior descriptive studies lacking statistical rigor, we applied a permutation-based null model to assess the reliability of network predictions. Subsequently, we employed gene set enrichment analysis (GSEA) to elucidate the core molecular mechanisms and biological pathways underlying each prescription. Through this integrative approach, we aim to provide a comprehensive understanding of the relationship between clinical evidence and molecular data. In addition, we present a new quantitative framework for analyzing complex herbal prescriptions. Accordingly, this study is positioned as a hypothesis-generating framework that aims to computationally explore the potential molecular mechanisms of NHPs, which should be validated through future experimental research.

## 2. Materials and Methods

### 2.1. Search Method

We analyzed clinically relevant papers on 56 herbal prescriptions registered as NHPs in Korea since the 1990s. English searches were conducted in PubMed and the Cochrane Library, considering different English names for the same prescriptions in Korea, China, and Japan.

- For 30 prescriptions, the English names provided by OASIS were used for English searches [6].
- For the remaining prescriptions, English names from papers listed on OASIS were used.
- For all 56 prescriptions, searches of KMBASE and OASIS were conducted in Korean.

### 2.2. Criteria for Excluding Prescriptions (Figure 1)

Prescriptions were excluded based on the following criteria:

- No papers found on OASIS or KMBASE.
  The following four prescriptions were excluded due to the absence of relevant literature in both OASIS and KMBASE: Dang-gwi-yeon-gyo-eum, Dang-gwi-yuk-hwang-tang, Sam-ho-jak-yak-tang, and Seung-yang-bo-wi-tang were excluded.
- Prescriptions with fewer than 10 non-duplicate papers across the OASIS, KMBASE, PubMed, and Cochrane databases.

Out of 52 prescriptions, 27 were excluded, and 25 were selected.

### 2.3. Criteria for Excluding Papers (Figure 1)

Papers were excluded based on the following criteria:

- Papers published before 1990.
- Papers in which the prescription name or specific disease were not mentioned in the title.
- Study protocols.
- Studies involving modified prescriptions, combined formulas, or combined with other prescriptions.
- Animal experiments instead of clinical trials.
- Studies reporting comparisons with other prescriptions, where the prescription of interest serves as the control.
- Statistically insignificant effects of the prescription in studies that compared the effects of various drugs on a specific disease.

Prescriptions were also excluded if fewer than five studies remained for a prescription after applying the above criteria.

### 2.4. Construction of the Prescription-Herb-Ingredient-Gene-Disease Network

**Figure 2:**
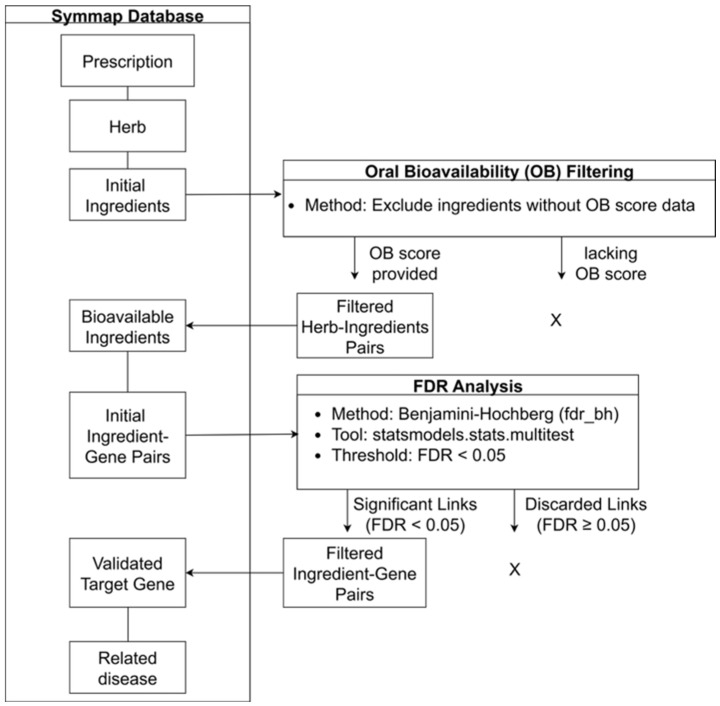
Overview of the sequential data filtering and network construction pipeline. This flowchart illustrates the pipeline for constructing the multi-layer network. The process starts with data extraction for prescriptions, herbs, and ingredients from the SymMap database, followed by a two-step filtering process based on oral bioavailability (OB) scores and a stringent False Discovery Rate (FDR) correction (Benjamini–Hochberg, FDR < 0.05). This systematic methodology ensures that only high-confidence interactions are carried forward for the final network assembly and subsequent disease prediction. This workflow demonstrates the reproducibility and transparency of our data-driven network construction, a key step for integrating clinical and molecular layers. X indicates filtered-out items.

The multilayer therapeutic network was constructed followed a systematic, multi-step process, which is visually detailed in Figure 2.

Data were first acquired from the SymMap database, from which we extracted the foundational layers of the network—prescriptions, their constituent herbs, and all associated chemical ingredients.

Subsequently, we implemented a sequential, two-step filtering protocol to refine this initial dataset, ensuring both the physiological relevance and statistical robustness of the components. First, a biological relevance screening was performed based on oral bioavailability (OB). To focus on compounds with a higher potential for in vivo activity, ingredients lacking an OB score in SymMap were excluded.

For the remaining bioavailable ingredients, we established statistically significant links to their target genes by addressing the challenge of multiple hypothesis testing. We applied the Benjamini–Hochberg (BH) procedure to control the false discovery rate (FDR), using the multipletests function from Python’s statsmodels library (v0.14.2) to adjust the raw *p*-values. Only ingredient–gene associations with an FDR-adjusted *p*-value of less than 0.05 were retained as validated interactions.

Finally, these rigorously filtered components were assembled into the final four-layer network, connecting prescriptions, herbs, bioavailable ingredients, and their validated target genes to associated diseases. The workflow was implemented in Python (v3.12.7). Data manipulation was managed with Pandas (v2.2.2), network construction and analysis were performed using the NetworkX library (v3.3), and final visualizations were generated with Matplotlib’s Pyplot interface (v3.9.2).

For network visualization, to improve interpretability while preserving the full analytical structure of the network, we adopted a layered visualization strategy. The complete network, including all nodes and edges, was used for all computational analyses. However, for figure generation, we displayed only the top 1% of nodes ranked by betweenness centrality within each node class (herb, ingredient, target, and disease). When the total number of nodes was large and this 1% selection exceeded 50 nodes, the visualization threshold was reduced to the top 0.5% to preserve visual clarity. This step reduced visual congestion. Betweenness centrality was chosen because it highlights nodes that mediate key connectivity pathways. This allows the structural backbone of the network to be preserved while minimizing visual complexity. Importantly, this reduction was used only for visualization and did not affect any statistical or pathway results.

Nodes were then arranged in a stacked layout by node class (Herb, Ingredient, Target, and Disease) using Cytoscape (v3.10.4). This layout clarifies the hierarchical flow of interactions across the four biological layers. As with the node reduction, this layout adjustment was applied solely for visualization and did not alter the underlying network structure or analytical outcomes.

### 2.5. Statistical Validation Using a Null Model

To assess the statistical significance of the network’s predictions, we implemented a permutation-based null model analysis [7]. The objective was to determine whether the observed concordance between our network’s predicted diseases and the clinical evidence was significantly greater than that expected by random chance.

The process began with the definition of three key datasets. First, a “gold-standard” set of clinically validated indications for each of the 13 prescriptions was established from a systematic literature review. To add nuance, these indications were manually curated and classified as primary (direct symptoms or diseases targeted), secondary (related comorbidities or complications), or all (for prescriptions with a single, well-defined therapeutic use). Second, the “network-predicted” set for each prescription was defined as all nodes with a “disease” attribute extracted from its final network graph. Third, a “background universe” was compiled from all unique disease terms in the SymMap database to serve as the population for random sampling.

An empirical null distribution was generated for each prescription and indication category through a permutation test repeated 10,000 times. In each permutation, a random set of diseases was sampled without replacement from the background universe. A critical set was to ensure a fair comparison by keeping the size of this random set identical to the number of diseases predicted by the actual network for that prescription. The concordance for this random set was then calculated by measuring its percentage of overlap with the corresponding gold standard list. This iterative process resulted in a null distribution of 10,000 concordance rates, representing outcomes achievable purely by chance.

To determine statistical significance, an empirical *p*-value was calculated using the formula *p* = (C + 1)/(N + 1), where C is the count of permutations with concordance rate greater than or equal to the observed rate and N is the total number of permutations (10,000). The result was considered statistically significant if its *p*-value was less than 0.05. The entire analysis was implemented in Python (v3.12.7), utilizing the Pandas library (v2.2.2) and statsmodels (v0.14.2) for data handling and Matplotlib (v3.9.2) for visualizing the null distributions.

### 2.6. GSEA of Gene Ontology (GO) and Kyoto Encyclopedia of Genes and Genomes (KEGG) Pathways

To elucidate the underlying molecular mechanisms and biological pathways associated with each prescription, GSEA was performed using the cluster Profiler package (v4.16.0) in R (v4.5.1) [8].

First, a pre-ranked gene list was generated for each of the 13 prescriptions. The ranking metric for each gene was calculated by weighing the inferred evidence scores from the SymMap database with the corresponding herb’s dosage in grams (g) within the prescription. Specifically, for a single gene, scores derived from all relevant herbs within the prescription were summed to reflect the polypharmacological effect of the herbal formula. This process resulted in a single comprehensive score for each unique gene per prescription.

Subsequently, gene symbols were converted to Entrez Gene IDs, the standard identifier used by the annotation database org.Hs.eg.db. The final ranked list was created by ordering all genes in descending order based on this comprehensive score.

GSEA was performed separately for GO, biological process (BP) terms, and KEGG pathways. For GO analysis, the gseGO function was utilized to identified enriched terms within the BP ontology. The analysis was configured to consider only gene sets with a minimum of 10 and a maximum 500 genes (minGSSize = 10, maxGSSize = 500). We focused on positively enriched gene sets (scoreType = “pos”) with a nominal *p*-value cutoff of 0.05 (pvalueCutoff = 0.05). Similarly, for KEGG pathway analysis, the gseKEGG function was applied for the human organism (organism = ‘hsa’) using the same parameters.

For all analyses, the resulting *p*-values were adjusted for multiple comparisons using the BH procedure to control the FDR.

All parameters, including gene set size limits, enrichment mode, and adjusted *p*-value thresholds, were defined a priori and are fully reported here to ensure methodological reproducibility and transparency.

This configuration emphasizes system-level, polypharmacological insights rather than isolated single-target effects.

## 3. Results

### 3.1. Primary Indications of NHPs

To identify the relationships between NHPs and diseases derived from clinical studies and the herbal medicine–molecular mechanism network, we investigated the main indications of 56 NHPs based on information provided by the Ministry of Health and Welfare of Korea [9]. The primary indications of 56 NHPs are available in Supplementary Table S1.

### 3.2. Selection of Clinically Relevant NHPs for Comparative Analysis

**Figure 3:**
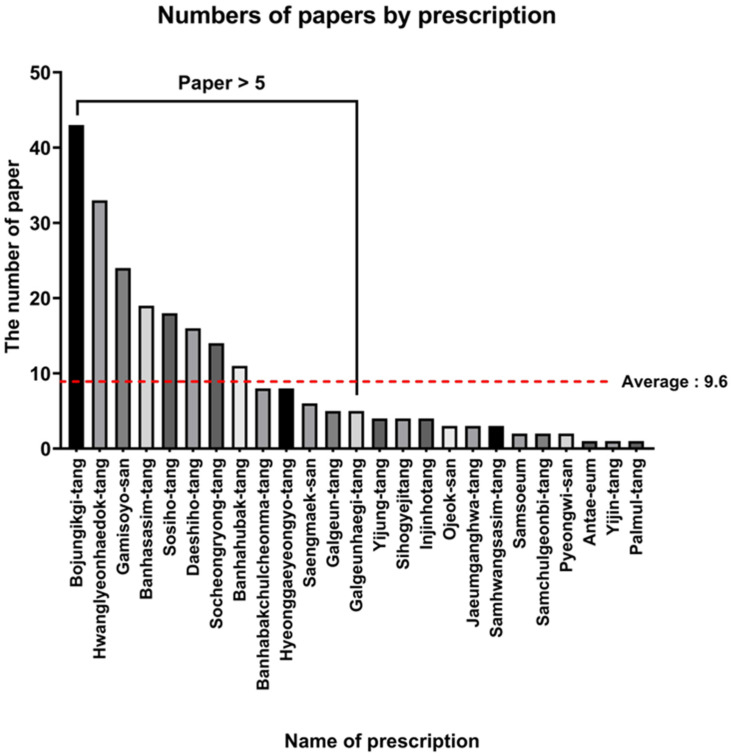
Number of studies identified for each prescription. This bar chart displays the number of clinical studies that met the final inclusion criteria for each of the 13 selected prescriptions. An initial screening of 56 NHPs was narrowed down through a systematic process based on literature availability and study quality. The significant reduction from 56 to 13 prescriptions highlights the scarcity of robust clinical data and underscores the rigorous criteria applied in this study to ensure that only prescriptions with a sufficient evidence base were included for analysis. This variability in study counts also suggests that the statistical power for subsequent validation may differ across prescriptions. The red dashed line indicates the overall average number of studies.

A literature search for clinical studies related to these NHPs was conducted. Initially, among the 56 NHPs, 25 were selected by excluding those with no relevant clinical studies or fewer than 10 published articles. Among the 25 NHPs initially selected, 13 prescriptions with five or more related research articles were ultimately included in further analysis (Figure 3).

### 3.3. Herb-Based Reconstruction of Prescription Networks

**Figure 4:**
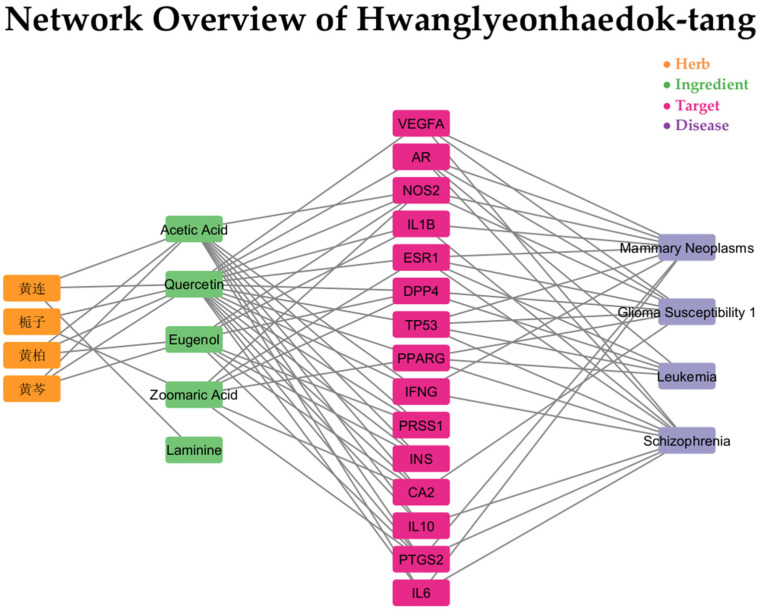
Network overview of Hwanglyeonhaedok-tang. The network illustrates the complex relationships between the prescription’s constituent herbs (orange), ingredients (green), molecular targets (pink), and associated diseases (purple). As the complete network is highly dense and visually complex, this figure displays a representative sub-network to enhance interpretability. This sub-network was generated by filtering for the top 1% of nodes ranked by betweenness centrality within each node class (see Section 2.4). The nodes were subsequently arranged in a stacked layout by class to clarify the hierarchical flow of interactions from herbs to diseases. This visualization strategy highlights the polypharmacological nature of NHPs, illustrating how multiple ingredients can converge on shared targets implicated in a broad spectrum of pathologies. This node reduction and specific layout are applied exclusively for visualization purposes. Non-English herb names appearing in the figure are traditional Chinese medicine terms written in Chinese characters: 黄连 (Coptidis Rhizoma), 黄芩 (Scutellariae Radix), 黄柏 (Phellodendri Chinensis Cortex), and 栀子 (Gardeniae Fructus).

**Figure 5:**
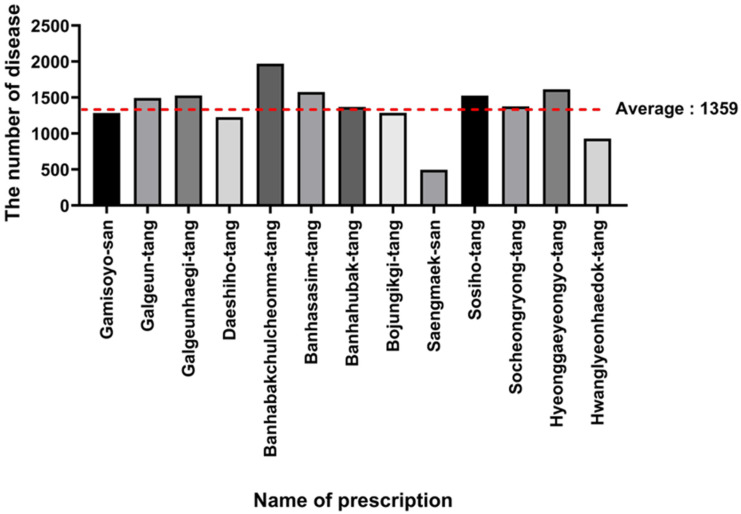
Number of diseases associated with each prescription network. This bar graph displays the number of potential diseases associated with each of the 13 prescriptions, as predicted by the network model. The analysis revealed that each prescription was linked to a vast number of pathologies, with an average of 1359 diseases per formula. This result highlights the extensive number of pathologies potentially associated with each formula. This finding supports the interpretation of NHPs as having polypharmacological properties and reinforces the utility of the network model as an exploratory tool for hypothesis generation rather than for single-disease prediction. The red dashed line indicates the overall average number of diseases.

To infer potential disease indications for each prescription by linking herbal networks to prescriptions, a network connecting prescriptions with herb–disease associations derived from the SymMap database was constructed and visualized. This network was visualized in four layers, including constituent herbs, constituent ingredients, target genes, and related diseases, using Python, NetworkX and Cytoscape, as shown in Figure 4. The networks for the remaining 13 prescriptions are shown in Supplementary Figure S1. Construction of this network revealed the average number of diseases identified as potentially applicable by the network for each prescription was 1359, as detailed in Figure 5. In addition, for each prescription, the top 20 most frequently represented diseases identified as potentially applicable through the network analysis are summarized and compared with existing indications (Table 1). If a disease matched an indication reported in clinical studies, it was marked as “(Clinical)”; if it matched the main indication of the prescription, it was marked as “(Main)”. When both clinical evidence and the main indication aligned, the disease was labeled as “(Clinical, Main)” next to its name. As a result, 6 of the 13 prescriptions (Galgeun-tang, Daeshiho-tang, Banhasasim-tang, Bojungikgi-tang, Saengmaek-san, and Hwanglyeonhaedok-tang) were consistent with clinical studies, main indications, or both. These findings suggest that the diseases predicted through network analysis for each prescription may be meaningfully associated with real-world clinical use and traditional indications.

Table 1 lists the 20 diseases most strongly associated with each prescription. Diseases confirmed in clinical studies or main indications are shown in bold and labeled as “Clinical” or “Main”, respectively.

### 3.4. Statistical Validation of Network Predictions

To validate our network’s predictions against random chance, a permutation-based null model analysis was performed for each of the 13 prescriptions. The 13 prescriptions showed statistically significant concordance (*p* < 0.05) in at least one indication category.

The predictive performance was highly significant (*p* < 0.001) for the primary indications of several prescriptions, including Bojungikgi-tang (*p* = 0.0001), Sosiho-tang (*p* = 0.0001), Banhahubak-tang (*p* = 0.0003), and Hwanglyeonhaedok-tang (*p* = 0.0007), as well as for the all indication category of Daeshiho-tang (*p* = 0.0001) and Banhasasim-tang (*p* = 0.0001). Some prescriptions showed differential performance; for instance, Saengmaek-san was significant for secondary (*p* = 0.0017) but not primary indications (*p* = 0.1309).

**Figure 6:**
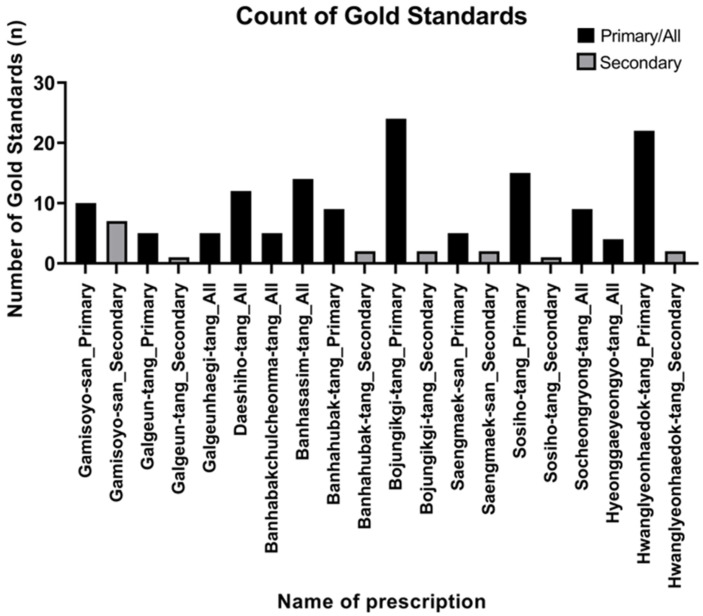
Number of gold standard indications for each prescription. This bar chart quantifies the number of unique clinical indications (n) curated from the literature for each of the 13 prescriptions, which served as the “gold standard” for validating the network’s predictions. These indications were categorized as primary, secondary, or all, forming the baseline for the null model analysis. The variability in the number of curated indications across prescriptions reflects the differing volumes of available clinical research and directly impacts the statistical power of the subsequent validation analysis. Black bars represent primary/all indications, while gray bars represent secondary indications. Black bars represent the number of primary/all gold standard indications, while gray bars represent the number of secondary gold standard indications. This highlights the importance of data availability in determining the reliability of network-based validation outcomes.

**Figure 7:**
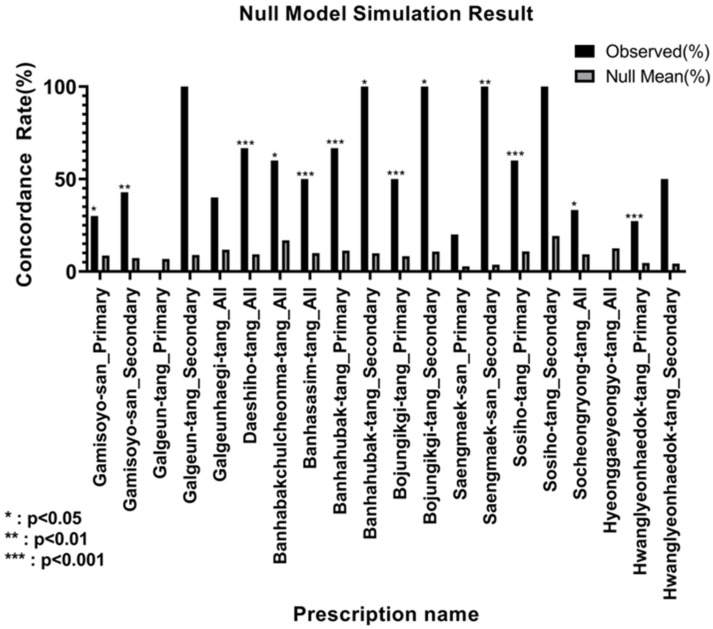
Statistical validation of network predictions using a null model. This bar plot compares the observed concordance rate (black bars) with the mean concordance rate from the null distribution (gray bars) for each prescription and indication type. The *y*-axis represents the concordance rate (%), and the *x*-axis lists the prescription along with its analyzed indication category (primary, secondary, or all). Error bars on the gray bars represent the standard deviation of the null distribution. Asterisks denote the level of statistical significance based on the empirical *p*-value. Overall, these results provide statistical support for the clinical relevance of the network’s predictions, suggesting that the systems-level approach of this study effectively captures meaningful biological information beyond random chance.

Conversely, predictions for several prescriptions did not reach statistical significance. Specifically, for Galgeun-tang, the concordance was 0.00% for primary indications (*p* = 1.0000) and 100.00% for secondary indications (*p* = 0.0894). Similarly, Galgeunhaegi-tang (40.00% concordance, *p* = 0.1060) and Hyeonggaeyeongyo-tang (0.00% concordance, *p* = 1.0000) did not show significant results. Notably, for the secondary indications of Galgeun-tang and Sosiho-tang, the observed concordance was 100%, but the *p*-values were not significant (*p* = 0.0894 and *p* = 0.1920, respectively), as the sample size was small (n = 1). Figure 6 specifies the number of gold standard indications (n) used for each analysis (details for all gold standard indications and their corresponding references are presented in Supplementary Table S2), and the corresponding null model distribution plots are shown in Figure 7.

### 3.5. GSEA Results of 13 NHPs

Through the GSEA of 13 different NHPs, common mechanisms of action and unique, specific mechanisms for each composite formula were identified via GO and KEGG pathway analysis.

First, three main axes of action were commonly observed across the 13 NHPs. (1) Hormone and Metabolic Regulation Axis: GO terms related to ‘response to hormone’ were commonly found in all 13 prescriptions. Associated KEGG pathways, such as those for hormone signaling and metabolic regulation, were also significantly detected. (2) Cell–cell Signaling (Nervous System) Axis: GO terms like ‘cell–cell signaling’ and ‘synaptic signaling’ were identified in 11 out of the 13 prescriptions. Furthermore, the KEGG pathway ‘Neuroactive ligand-receptor interaction’ and related synapse/neurotransmission pathways were significant in nine of the prescriptions. (3) Regulation of Cell Proliferation and Death Axis: The GO term ‘regulation of cell population proliferation’ was commonly detected in 10 out of the 13 prescriptions. In all prescriptions, a recurring association with sub-modules related to the cell cycle and survival signals within the KEGG ‘Pathways in cancer’ was observed. While ‘Apoptosis’ as a separate KEGG entry was not always independently significant, a gene overlap with ‘Pathways in cancer’ was observed.

Next, the analysis of the specific action axes for individual NHPs revealed the following unique GO and KEGG pathways for each. Bojungikgi-tang: GO terms related to protein synthesis and transcription (GO:0045893, GO:1902680) were specifically detected. The associated KEGG pathways included ‘AGE-RAGE signaling in diabetic complications (hsa04933)’, ‘Alzheimer’s disease (hsa05010)’, and ‘Lipid and atherosclerosis (hsa05417)’. Galgeun-tang: GO terms related to synapse and neural signaling (GO:0007268, GO:0099536, GO:0007267) were prominent. Specific KEGG pathways were related to infectious diseases, namely ‘Kaposi sarcoma-associated herpesvirus infection (hsa05167)’ and ‘Leishmaniasis (hsa05140)’. Saengmaeksan: GO terms related to damage repair and maintenance of cellular homeostasis (GO:0035556, GO:0065008) were discovered. Associated KEGG pathways included the specific immune pathway ‘IL-17 signaling (hsa04657)’ and pathways related to tissue damage, such as ‘Pathways of neurodegeneration—multiple diseases (hsa05022)’ and ‘Alzheimer disease (hsa05010)’. Hwangryeonhaedok-tang: GO terms related to responses to chemical substances and stress (GO:0070887, GO:1901701, GO:1901700, GO:0009719, GO:0035556) were characteristically observed. In the KEGG analysis, only ‘Pathways in cancer (hsa05200)’ was detected within the level of significance. Hyeonggae-Yeongyo-tang: GO terms related to the regulation of stress and cell proliferation (GO:1901701, GO:1901699, GO:0042127, GO:0008283, GO:0009719) were significant. In its KEGG pathways, ‘Pathways in cancer (hsa05200)’ and ‘Neuroactive ligand-receptor interaction (hsa04080)’ were observed. Banhasasim-tang: GO terms related to neural and endocrine regulation (GO:0009725, GO:0007267, GO:0009719, GO:1901701) were detected. Specific KEGG pathways identified were ‘Serotonergic synapse (hsa04726)’ and ‘Retrograde endocannabinoid signaling (hsa04723)’. Sosiho-tang: GO terms related to broad-spectrum system regulation (GO:0007267, GO:0071495, GO:0009725, GO:1901701) were detected, with overlapping significance in various system-related KEGG pathways (e.g., ‘Pathways in cancer (hsa05200)’).

The complete GSEA results for all 13 prescriptions, including all significantly enriched GO terms and KEGG pathways (FDR < 0.05), are provided in Supplementary Table S3.

## 4. Discussion

In this study, we evaluated the efficacy of 56 NHPs by integrating clinical research with systems biology information. We performed a comparative analysis between diseases identified from clinical studies and those from public databases. We searched for clinical papers on the 56 NHPs using domestic and international research search engines such as KMBASE, PubMed, and the Cochrane Review, and found that extensive clinical research had been conducted on 13 of these prescriptions. Using these data, we utilized the SymMap database to identify the 20 diseases most likely related to each prescription. The resulting dataset was used to construct network visualizations.

Subsequently, network visualizations for the 13 NHPs were performed using NetworkX and Pyplot in Python, and through these networks, the diseases to which each prescription could be applied were analyzed. In addition, the network analysis results for the 13 prescriptions were compared with the corresponding clinical papers. To verify the non-randomness of the network predictions, a permutation-based null model analysis was conducted for each of the 13 prescriptions. Finally, to identify the underlying molecular mechanisms and biological pathways associated with each prescription, GSEA was performed using the clusterProfiler package (v4.16.0) in R (v4.5.1).

The results revealed a nuanced relationship between the ranking-based prediction of diseases and the broader disease landscape inferred from the network model. When the top 20 diseases with the highest prediction frequency were directly compared with actual clinical cases, the concordance rate was low. As detailed in Table 1, no matches were found within this top list for seven of the 13 prescriptions. These findings suggest that the top-ranked diseases with the highest prediction frequency from the network model may not operate in a manner that precisely identifies clinical outcomes.

However, this low concordance rate in the top-ranked predictions does not necessarily undermine the clinical validity of the model. Such discrepancies likely stem from the keyword-based matching of disease names. For instance, clinical studies often describe symptoms—such as heartburn or nausea—rather than formal disease entities like gastroesophageal reflux disease. This difference hinders direct comparison and suggests the need for a broader level of interpretation.

Furthermore, these analyses do not necessarily indicate that the network predictions are biologically irrelevant. The large number of predicted diseases per prescription aligns with the polypharmacological characteristics of multi-herb formulas, in which multiple compounds interact with pleiotropic targets affecting diverse physiological processes. Additionally, all ingredient–target associations were filtered using oral bioavailability constraints and multiple-testing correction (FDR < 0.05), which may help reduce random statistical noise.

The overlap observed in predicted disease profiles across prescriptions may therefore be interpreted as reflecting the shared modulation of core regulatory axes, rather than non-specific or indiscriminate prediction. Consistent with the GSEA results (Section 3.5), these shared axes include (1) hormone–metabolic homeostasis, (2) neural cell–cell signaling networks, and (3) regulation of cell proliferation. In this context, the model may be characterizing system-level physiological tendencies rather than one-to-one disease specificity.

To overcome this limitation and evaluate the overall clinical validity of the network predictions, we introduced a null model analysis. The purpose of this analysis was to determine whether the complete list of diseases predicted by the network was more significantly associated with the known clinical effects of the prescription (classified as primary, secondary, or all indications) than a randomly generated list of diseases. As shown in Figure 7, the results were statistically significant for 10 out of the 13 prescriptions. In particular, prescriptions, such as Bojungikgi-tang (*p* = 0.0001), Sosiho-tang (*p* = 0.0001), and Banhahubak-tang (*p* = 0.0003), demonstrated very high statistical significance (*p* < 0.001), which suggests that the network captures clinically valuable biological information.

Taken together, these findings indicate that the network model is better understood as an exploratory framework for generating hypotheses about shared regulatory themes and potential therapeutic ranges of multi-herb prescriptions. Accordingly, the predictions in this study should be interpreted as hypothesis-generating rather than confirmatory.

Most NHPs exhibit a polypharmacological nature. They potentially modulate broad regulatory networks involving hormones, neural signaling, and cell proliferation to modulate multiple targets simultaneously. As such, they may function as potential homeostasis regulators rather than focusing on a single disease target. This mechanism may help restore physiological balance disrupted by complex diseases [16]. Specifically, the simultaneous regulation of neuroactive ligand–receptor and hormonal response pathways can be understood through the modern concept of the gut–brain axis. In this context, gut microbiota can metabolize external substances, such as NHPs, to directly produce neuroactive ligands (neurotransmitters) or modulate the secretion of gut hormones. This process may provide a molecular basis for how NHPs influence systemic neuroendocrine regulation [17].

Additionally, we observed that the ‘Pathways in cancer’ pathway was commonly implicated across all NHPs. We identified the sub-mechanisms that NHPs commonly regulate by cross-analyzing the core enrichment genes that contributed to the pathway’s significance. As a result, a total of 48 genes were found to be common to all 13 prescriptions. This gene group included *EGFR* and *FGF*, which are involved in growth factor signaling; the *CDK* and *Cyclin* families, which directly regulate the cell cycle; and key intracellular signaling mediators, such as *PI3K* and *MAPK*. These findings may suggest that NHPs do not act indiscriminately throughout the cancer-related signaling network. Instead, they appear to intervene selectively at three key stages: (1) receiving external growth stimuli, (2) processing intracellular signal transduction, and (3) committing to cell division. Accordingly, NHPs may help maintain tissue homeostasis and ameliorate the disease microenvironment by regulating, from multiple angles, the proliferation signals that are aberrantly activated under stress conditions such as chronic inflammation [18,19].

The clinical efficacy of each NHP can also be interpreted in greater depth by linking it to the specific molecular pathways identified in this study. For instance, Bojungikgi-tang was associated with the regulation of protein synthesis and transcription (e.g., GO:0045893). This finding suggests that its pharmacological actions may extend beyond the classical role of replenishing energy (qi) and instead encompass the modern concept of cellular regeneration [20]. Moreover, the regulation of the Advanced Glycation End-products (AGE)–Receptor for Advanced Glycation End-products (RAGE) signaling pathway, which is critically involved in aging and metabolic stress, indicates that Bojungikgi-tang may confer protective effects by attenuating the progression of chronic degenerative diseases [21].

In the case of Galgeun-tang, its simultaneous regulation of pathways related to infectious diseases and synaptic signaling is an interesting finding that supports a dual-mechanism hypothesis for Galgeun-tang’s efficacy. The prescription may contribute to treatment not only by promoting immune responses, but also by directly modulating nervous system-mediated symptoms of infection, such as pain and myalgia [22].

This study also suggested that distinct NHPs can act through different molecular pathways to achieve similar anti-inflammatory outcomes. Saengmaeksan may act mainly through IL-17 signaling and tissue repair [23]. In contrast, Hwangryeonhaedok-tang seems to control early inflammatory responses triggered by chemical stress [24]. In contrast, Hyeonggae-Yeongyo-tang seems to focus on controlling the pathological cell proliferation that follows the inflammatory phase [25].

Meanwhile, our analysis identified the ‘Serotonergic synapse’ and ‘Endocannabinoid signaling’ pathways in association with Banhasasim-tang. This suggests that its effectiveness for stress, anxiety, and gastrointestinal dysfunction may be linked to a neuro-endocrine homeostatic mechanism, which is often mediated by the gut–brain axis [26,27].

Finally, Sosiho-tang potentially indicated broad pathway regulation lacking specificity for any single signaling axis. This suggests that it may function as a systemic regulator that restores homeostasis by resolving complex imbalances across multiple physiological systems, such as the immune, metabolic, and nervous systems. Recent studies based on network pharmacology have similarly shown that TCM acts in a multi-component, multi-target, and multi-pathway manner to modulate the immune microenvironment in complex diseases such as cancer. This supports the notion that complex prescriptions like Sosiho-tang have the potential to restore systemic homeostasis [28].

### 4.1. Broader Applicability and Future Implications of the Framework

The integrated framework validated in this study offers insights that extend beyond the 13 NHPs, holding significant implications for the future of TKM research and its clinical application.

First, in the field of drug discovery, this approach can function as a hypothesis-generation engine. Rather than screening compounds randomly, our framework can guide the development of novel polypharmacological drugs based on specific, validated mechanisms. For example, leveraging the common homeostasis regulatory mechanism identified in this study, one could design new therapies targeting complex, chronic, multi-system diseases. Furthermore, specific findings, such as the link between Banhasasim-tang and the gut–brain axis via serotonergic and endocannabinoid signaling, provide a direct rationale for developing new therapeutics for stress-related gastrointestinal and psychiatric disorders.

Second, from the perspective of clinical decision-making, this framework provides a scientific lens through which to understand and rationalize the polypharmacological effects of TKM formulas. Rather than relying solely on traditional indications, clinicians can use our mechanistic findings to make more nuanced decisions. For example, our GSEA suggested that different prescriptions with similar anti-inflammatory effects operate through distinct molecular pathways. Saengmaeksan appears to target a specific immune route (IL-17 signaling) and tissue repair, making it potentially suitable for inflammation involving tissue damage. In contrast, Hyeonggae-Yeongyo-tang appears specialized in controlling pathological cell proliferation that can occur after an inflammatory phase, suggesting its use at a different stage of the condition. This level of mechanistic detail offers a rational basis for selecting a specific NHP based on the patient’s underlying biological state, moving beyond a one-size-fits-all approach and enhancing the precision of traditional medicine.

Finally, in terms of health policy, this methodology provides a method of generating scientific evidence to support the use of traditional medicines. By translating the principles of TKM prescriptions into the language of molecular biology, it can supply the evidence base needed for policy decisions, such as those concerning health insurance coverage or integration into national healthcare systems. Ultimately, this study can serve as a reproducible template for modernizing the research and application of complex natural product-based medicines.

### 4.2. Limitations of the Study

There are certain limitations that should be considered when interpreting the findings of this study. First, clinical research in TKM remains markedly insufficient. As of August 2024, a keyword search for ‘clinical’ in the Korean Citation Index yielded 18,926 articles related to Western medicine but only 2196 related to TKM. This stark discrepancy highlights the paucity of accessible TKM clinical research, which consequently limited our analysis to 13 prescriptions out of the initial 56 NHPs. As a result, the reduced sample size may constrain the generalizability of these findings to the broader field of TKM [29].

Second, most clinical literature lacks documentation of TCM pattern (syndrome). Many analyzed studies did not include information on syndrome classification, which is fundamental for diagnosis and prescription in TCM practice. This omission made it difficult to interpret the results in a manner that supports syndrome-based clinical decision-making. The lack of such information may be partly due to the CASE guidelines for clinical case reports [30], which do not explicitly recommend including TCM syndrome. Consequently, we were unable to assess the correlation between TCM patterns and modern disease classifications, thereby limiting the potential for cross-systemic insights between TCM and Western medical frameworks.

Third, discrepancies between the Korean Pharmacopoeia and the Chinese databases used in this study further limited the data analysis. For instance, *Massa Medicata Fermentata*, a component herb of Banhabakchulcheonma-tang, was not included in the Chinese database, making it impossible to incorporate this herb into the network analysis. This discrepancy suggests that current databases do not comprehensively cover all traditional medicinal materials used across different countries, which may restrict the interpretation and applicability of the study’s findings.

Fourth, the inclusion of only 13 prescriptions introduces the possibility of sampling bias, limiting the generalization of our conclusions to all multi-component natural products. These 13 formulas were selected based on the availability of sufficient clinical evidence and standardized compositional data, which ensured analytical consistency but inevitably reduced the sample scope. Future studies should validate and expand upon these findings using larger datasets and broader prescription coverage.

Fifth, while the computational predictions offer valuable insights, they require subsequent experimental validation to substantiate their reliability and translational potential. The proposed mechanisms are data-driven hypotheses that must be empirically verified through future experimental studies. This distinction underscores that the findings of this work serve primarily as hypothesis-generating insights rather than definitive mechanistic conclusions. Nevertheless, the permutation-based statistical validation and the full transparency of the shared datasets provide a reproducible computational framework that can serve as a reliable foundation for subsequent empirical research.

Finally, it is important to recognize that some analyzed prescriptions, such as Galgeun-tang and Socheongryong-tang, contain *Ephedra herba*, a natural source of ephedrine alkaloids. Concerns regarding the safety of these compounds have led the U.S. Food and Drug Administration (FDA) to prohibit ephedrine-containing dietary supplements. However, the traditional medical context of use differs fundamentally from the scenarios that prompted these regulatory actions. The FDA’s concerns focused on long-term ephedrine use for weight loss or athletic enhancement, often with other stimulants. In contrast, Galgeun-tang is prescribed for short-term use for acute conditions such as the common cold. Moreover, within traditional formulations, *Ephedra* is used as part of a complex multi-herbal composition in which other herbs are believed to modulate its efficacy and mitigate potential toxicity. A systems biology approach, as implemented in this study, may help elucidate complex molecular interactions and composite pharmacological mechanisms.

## 5. Conclusions

This study presents a novel methodology that integrates TKM clinical research with a systems biology approach to explore and visualize the therapeutic indications for NHPs. Our analysis suggests that while the direct concordance between the top-ranked disease predictions of the network model and clinical cases was low, permutation-based null model analysis demonstrated that the overall set of network-predicted diseases was statistically significantly associated with known clinical indications. Furthermore, GSEA suggests that at the molecular level, NHPs may act as potential homeostatic regulators, modulating core biological axes, such as cell proliferation signaling, neural transmission, and hormonal responses.

The key finding is that the network model is more helpful in exploring broad therapeutic landscapes than for pinpointing single disease targets. Rather than serving as a tool for precise disease prediction, the model functions as an exploratory platform for uncovering previously unrecognized therapeutic possibilities and generating new scientific hypotheses. Nonetheless, this study is constrained by the limited availability of clinical data and the inherent limitations of existing databases. However, the computationally validated framework presented herein moves beyond a reinterpretation of past knowledge to offer concrete directions for future research.

This approach can contribute to three main areas. First, it can help in designing precision-medicine-based clinical trials. Specific molecular pathways identified through GSEA (e.g., IL-17 signaling, serotonergic signaling) can be used as biomarkers to stratify patient populations that are most likely to respond to a given prescription, thereby increasing the success rate of clinical trials. Second, it can help in developing novel polypharmacological therapies. The core mechanisms identified in this study, such as homeostasis regulation, can serve as targets for designing new combination drugs that may influence multiple disease systems simultaneously. Finally, this framework can function as a standardized template for evaluating the efficacy and mechanisms of other, less-studied complex natural medicines. Through these efforts, our approach will serve as a critical stepping stone for strengthening the scientific basis of traditional medicine and enhancing its clinical applicability.

Nevertheless, these computational predictions remain hypothesis-generating and should be validated through further experimental studies.

## Figures and Tables

**Figure 1:**
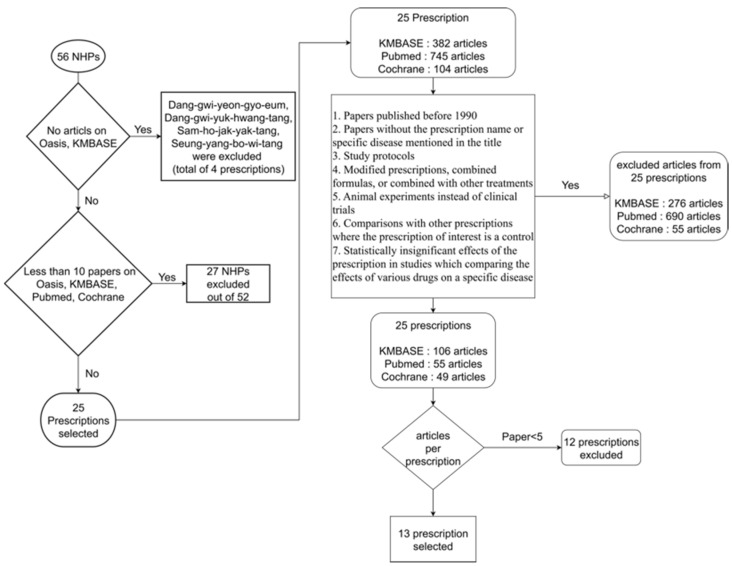
Overall flowchart of prescription and clinical study selection. Starting with 56 National Health Insurance Herbal Prescriptions (NHPs), a multi-stage filtering process was applied based on the availability of literature and the quality of clinical studies, as detailed in Section 2. This rigorous selection process was designed to identify a final cohort of 13 prescriptions with sufficient clinical evidence to ensure a robust, meaningful network-based pathway analysis. This selection framework ensures that the downstream network analysis reflects clinically grounded evidence rather than theoretical associations.

**Table 1:** Top 20 diseases potentially associated with each prescription network.

Prescription	Disease
Gamisoyo-san	Lung Cancer, Carcinoma of Lung. Lung Neoplasms, Schizophrenia, Non-Small Cell Lung Carcinoma. Diabetes Mellitus, Noninsulin-Dependent, Ovarian Neoplasm, Arthritis, Rheumatoid, Leukemia, Adenocarcinoma, Liver Cirrhosis, Experimental, Asthma, Aneurysm, Myocardial Infarction, Prostatic Neoplasms, Spinal Cord Ischemia, Diabetes Mellitus, Experimental, Malaria, Hepatoblastoma, Disorder of Eye
Galgeun-tang	Leukemia, Glioma, Lung Cancer, Kidney Neoplasm, Carcinoma of Lung, Lung Neoplasms, Adenocarcinoma, Non-Small Cell Lung Carcinoma, Schizophrenia, Melanoma, Prostatic Neoplasms, Astrocytoma, Hepatocellular Carcinoma, Myeloid Leukemia, Liver Carcinoma, Diabetes Mellitus, Non-Insulin-Dependent ^a^, Liver Cirrhosis, Experimental, Depressive Disorder, Lymphoproliferative Disorders, Ovarian Neoplasm
Galgeunhaegi-tang	Leukemia, Glioma, Lung Cancer, Adenocarcinoma, Lung Neoplasms, Carcinoma of Lung, Schizophrenia, Prostatic Neoplasms, Non-Small Cell Lung Carcinoma, Mammary Neoplasms, Diabetes Mellitus, Noninsulin-Dependent, Hepatocellular Carcinoma, Colorectal Neoplasms, Astrocytoma, Diabetes Mellitus, Experimental, Myeloid Leukemia, Liver Cirrhosis, Experimental, Hypertensive Disease, Kidney Neoplasm, Asthma
Daeshiho-tang	Adenocarcinoma, Lung Cancer, Carcinoma of Lung, Non-Small Cell Lung Carcinoma, Schizophrenia, Mammary Neoplasms, Glioma, Diabetes Mellitus, Noninsulin-Dependent, Hepatocellular Carcinoma, Prostatic Neoplasms, Liver Carcinoma, Ovarian Neoplasm, Malignant Neoplasm of Breast, Leukemia, Esophageal Neoplasms, Colorectal Neoplasms, Liver Cirrhosis, Experimental, Colorectal Cancer, Obesity ^b,†^, Malaria
Banhabakchulcheonma-tang	Schizophrenia, Diabetes Mellitus, Non-Insulin-Dependent, Mammary Neoplasms, Lung Cancer, Ovarian Neoplasm, Liver Cirrhosis, Experimental, Bipolar Disorder, Liver Carcinoma, Non-Small Cell Lung Carcinoma, Colorectal Neoplasms, Carcinoma of Lung, Hepatocellular, Carcinoma, Inflammatory Bowel Diseases, Alzheimer’s Disease, Asthma, Alcoholic, Intoxication, Chronic, Prostatic Neoplasms, Colorectal Cancer, Major Depressive Disorder, Lung Neoplasms
Banhasasim-tang	Mammary Neoplasms, Prostatic Neoplasms, Schizophrenia, Lung Cancer, Carcinoma of Lung, Hepatocellular Carcinoma, Liver Carcinoma, Animal Mammary Neoplasms, Leukemia, Liver Cirrhosis, Experimental, Diabetes Mellitus, Non-Insulin-Dependent, Colorectal Neoplasms, Ovarian Neoplasm, Non-Small Cell Lung Carcinoma, Malignant Neoplasm of Breast, Colitis (Main), Alzheimer Disease, Inflammatory Bowel Diseases, Depressive Disorder, Colorectal Cancer
Banhahubak-tang	Mammary Neoplasms, Schizophrenia, Leukemia, Lung Cancer, Diabetes Mellitus, Noninsulin-Dependent, Liver Cirrhosis, Experimental, Carcinoma of Lung, Lung Neoplasms, Alzheimer’s Disease, Astrocytoma, Major Depressive Disorder, Animal Mammary Neoplasms, Prostatic Neoplasms, Liver Carcinoma, Depressive Disorder, Melanoma, Diabetes Mellitus, Experimental, Alcoholic Intoxication, Chronic, Kidney Neoplasm, Colorectal Neoplasms
Bojungikgi-tang	Lung Cancer, Carcinoma of Lung, Schizophrenia, Prostatic Neoplasms, Diabetes Mellitus, Noninsulin-Dependent ^c^, Ovarian Neoplasm, Bipolar Disorder, Asthma ^d^, Liver Carcinoma, Non-Small Cell Lung Carcinoma, Liver Cirrhosis, Experimental, Lung Neoplasms, Hypertensive Disease, Mammary Neoplasms, Arthritis, Rheumatoid, Myocardial Infarction, Ovarian Cancer, Colorectal Neoplasms, Autistic Disorder, Adult Primary Hepatocellular Carcinoma
Saengmaek-san	Mammary Neoplasms, Prostatic Neoplasms, Renal Cell Carcinoma, Bipolar Disorder, Malaria, Disorder of Eye, Diabetes Mellitus, Noninsulin-Dependent ^e^, Induced Malaria, Insulin Resistance, Malaria, Cerebral, Ewings Sarcoma-Primitive Neuroectodermal Tumor (Primitive neuro-ectodermal tumor), Ewings Sarcoma, Arthritis, Rheumatoid, Mood Disorders, Schizophrenia, Asthma, Obesity, Nervous System Disorder, Ovarian Neoplasm, Mental Retardation
Sosiho-tang	Mammary Neoplasms, Prostatic Neoplasms, Lung Cancer, Schizophrenia, Carcinoma of Lung, Leukemia, Diabetes Mellitus, Non-Insulin-Dependent, Hepatocellular Carcinoma, Ovarian Neoplasm, Liver Carcinoma, Non-Small Cell Lung Carcinoma, Lung Neoplasms, Animal Mammary Neoplasms, Liver Cirrhosis, Experimental, Glioma, Colorectal Neoplasms, Alzheimer’s Disease, Disorder Of Eye, Obesity, Malignant Neoplasm of Breast
Socheongryong-tang	Schizophrenia, Lung Cancer, Carcinoma of Lung, Non-Small Cell Lung Carcinoma, Hepatocellular Carcinoma, Liver Carcinoma, Liver Cirrhosis, Experimental, Myocardial Infarction, Diabetes Mellitus, Non-Insulin-Dependent, Prostatic Neoplasms, Mammary Neoplasms, Bipolar Disorder, Esophageal Neoplasms, Alzheimer’s Disease, Depressive Disorder, Diabetes Mellitus, Experimental, Hypertensive Disease, Glioma, Ovarian Neoplasm, Leukemia
Hyeonggaeyeongyo-tang	Schizophrenia, Lung Cancer, Carcinoma of Lung, Diabetes Mellitus, Non-Insulin-Dependent, Prostatic Neoplasms, Non-Small Cell Lung Carcinoma, Asthma, Lung Neoplasms, Adenocarcinoma, Hypertensive Disease, Colorectal Neoplasms, Liver Cirrhosis, Experimental, Mammary Neoplasms, Diabetes Mellitus, Experimental, Colorectal Cancer, Hepatocellular Carcinoma, Myocardial Infarction, Arthritis, Rheumatoid, Bipolar Disorder, Ovarian Neoplasm
Hwanglyeonhaedok-tang	Mammary Neoplasms, Leukemia, Schizophrenia, Prostatic Neoplasms, Malignant Neoplasm of Breast, Breast Cancer, Colorectal Neoplasms, Glioma Susceptibility 1, Colorectal Cancer, Liver Cirrhosis, Experimental, Glioblastoma, Asthma †, Inflammatory Bowel Diseases, Lung Neoplasms, Non-Small Cell Lung Carcinoma, Hepatocellular Carcinoma, Liver Carcinoma, Hypertensive Disease, Prostate Cancer, Diabetes Mellitus, Noninsulin-Dependent ^f^

^a^—Matches an indication reported in clinical studies [10]. ^b^—Matches an indication reported in clinical studies [11]. ^c^—Matches an indication reported in clinical studies [12]. ^d^—Matches an indication reported in clinical studies [13]. ^e^—Matches an indication reported in clinical studies [14]. ^f^—Matches an indication reported in clinical studies [15]. †—Matches the main indication of the prescription.

## Data Availability

All datasets, Python and R code, Null model histograms, and raw GSEA result data used in this study have been uploaded to the following GitHub repository: https://github.com/jds4682/data_for_NHP_paper (accessed on 7 October 2025) and permanently archived at Zenodo (DOI: 10.5281/zenodo.17288021). The raw herbal-compound-target-disease association data were retrieved from the SymMap database, which is publicly available at http://www.symmap.org/. All other additional data supporting the findings of this study are included within the article and its Supplementary Materials.

## Supplementary Materials

The following supporting information can be downloaded at: https://www.mdpi.com/article/10.3390/life15111769/s1, Supplementary Table S1: Main indication of the 56 prescriptions; Supplementary Table S2: Detail for all gold standard indications; Supplementary Table S3: S3 GSEA Results for 13 NHPs; Supplementary Figures S1–S13: Overview of Networks.

## Abbreviations

The following abbreviations are used in this manuscript:

NHP	National Health Insurance Herbal Prescriptions
TKM	Traditional Korean Medicine
TCM	Traditional Chinese medicine
OASIS	Oriental Medicine Advanced Searching Integrated System
KMBASE	Korean Medical database
GSEA	Gene Set Enrichment Analysis
KEGG	Kyoto Encyclopedia of Genes and Genomes
OB	Oral Bioavailability
FDR	False Discovery Rate
GO	Gene Ontology
BP	Biological Process
BH	Benjamini–Hochberg
AGE	Advanced Glycation End-products
RAGE	Receptor for Advanced Glycation End-products
