# Supplementary material for: Network Analysis of Predicted Therapeutic Symptoms in National Health Insurance Herbal Prescriptions
Source: Life (Basel). 2025 Nov 18;15(11):1769. doi: 10.3390/life15111769 (PMC12654001; doi:10.3390/life15111769)
Supplement: Supplementary file 1 [file life-15-01769-s001.zip › life-3962368-supplementary/Supplementary figures_fixed.pdf]

**Overview of Networks**

Each network illustrates the relationships among constituent herbs, ingredients, molecular targets, and associated diseases. All networks use the same color scheme as Figure 4: herbs (orange), ingredients (green), molecular targets (pink), and diseases (purple).

Because the complete networks are highly dense and visually complex, representative subnetworks are displayed to enhance interpretability. These subnetworks were generated by filtering for the top 1% of nodes ranked by betweenness centrality within each node class (see Methods 2.4). When the total number of nodes was large and this 1% selection exceeded 50 nodes, the visualization threshold was reduced to the top 0.5% to preserve visual clarity.

Nodes were arranged in a stacked layout by class to clarify the hierarchical flow of interactions from herbs to diseases. This visualization strategy highlights the polypharmacological nature of NHPs, showing how multiple ingredients can converge on shared targets across a wide range of pathologies. Node reduction and the specific layout were applied exclusively for visualization purposes.

NHP, National Health Insurance herbal prescriptions

**Supplementary Figure S1.** Network Overview of Gamisoyo-san.

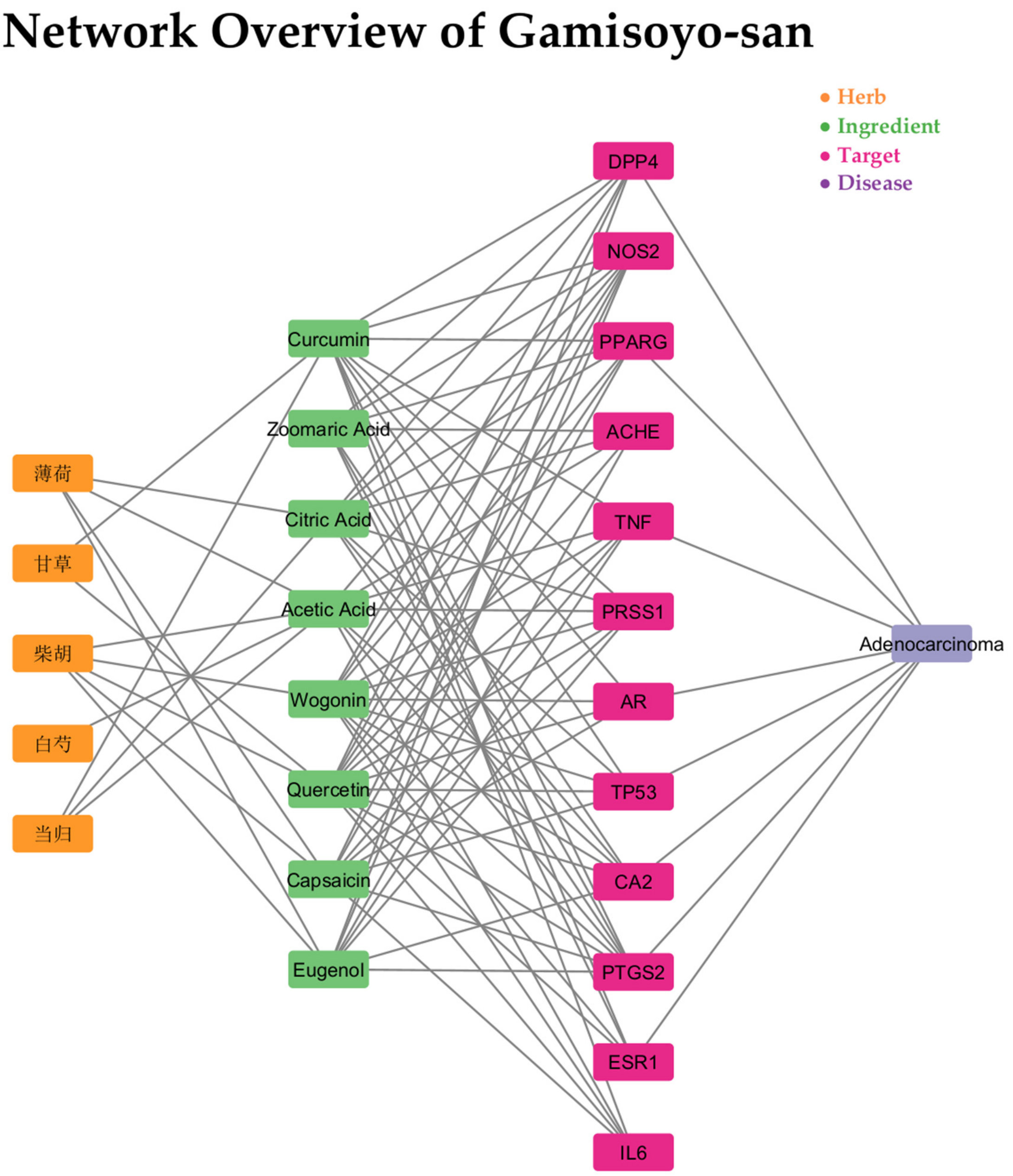


Below are the non-English herb names appearing in the figure. Each is provided with its standard Traditional Chinese Medicine name (Chinese characters) and Latin pharmaceutical name. 薄荷 (Menthae Haplocalycis Herba), 甘草 (Glycyrrhizae Radix et Rhizoma), 柴胡 (Bupleuri Radix), 白芍 (Paeoniae Radix Alba), 当归 (Angelicae Sinensis Radix)

**Supplementary Figure S2.** Network Overview of Galgeun-tang.

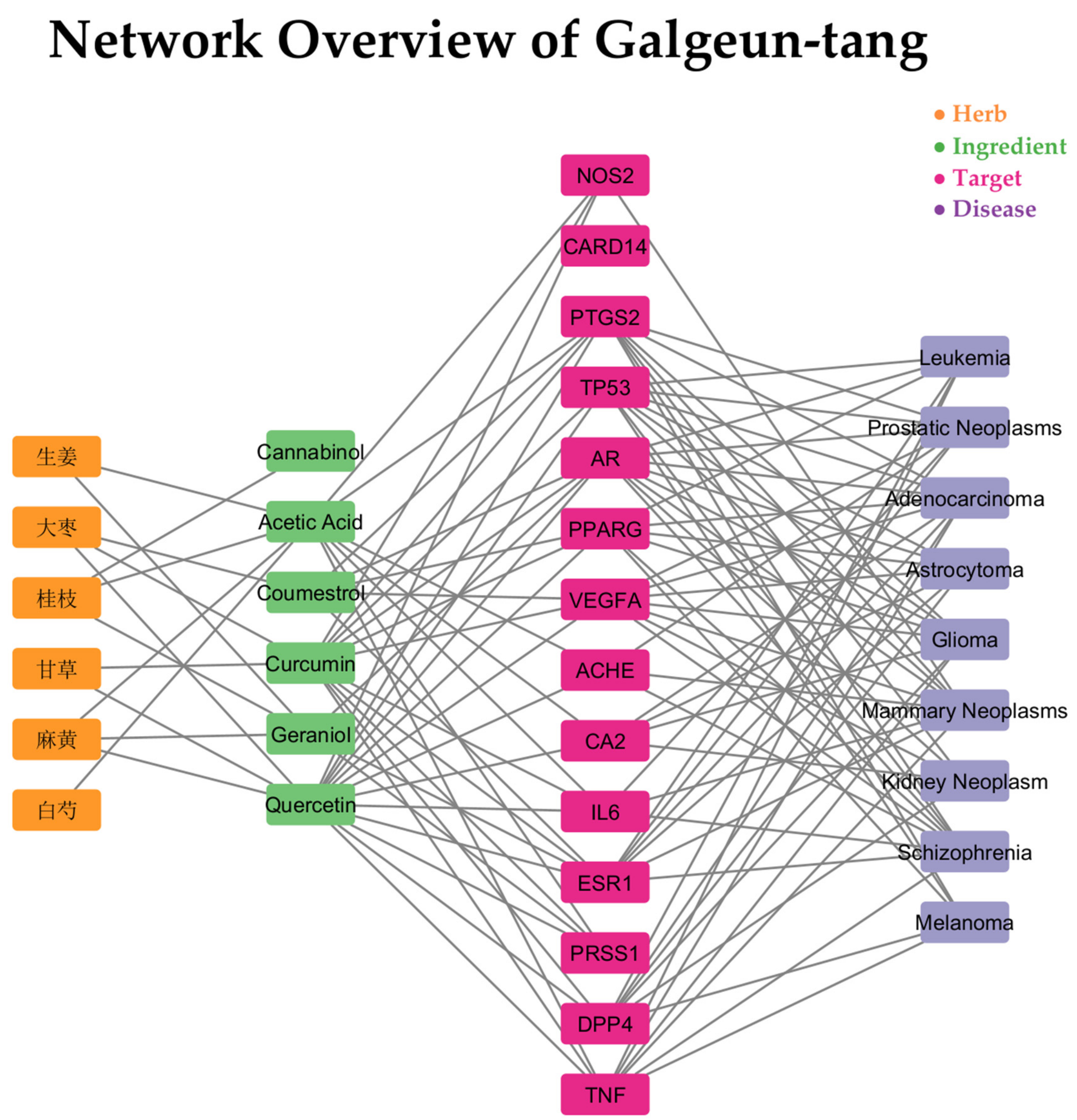


Below are the non-English herb names appearing in the figure. Each is provided with its standard Traditional Chinese Medicine name (Chinese characters) and Latin pharmaceutical name. 生姜 (Zingiberis Rhizoma Recens), 大枣 (Jujubae Fructus), 桂枝 (Cinnamomi Ramulus), 甘草 (Glycyrrhizae Radix et Rhizoma), 麻黄 (Ephedrae Herba), 白芍 (Paeoniae Radix Alba)

**Supplementary Figure S3.** Network Overview of Galgeunhaegi-tang.

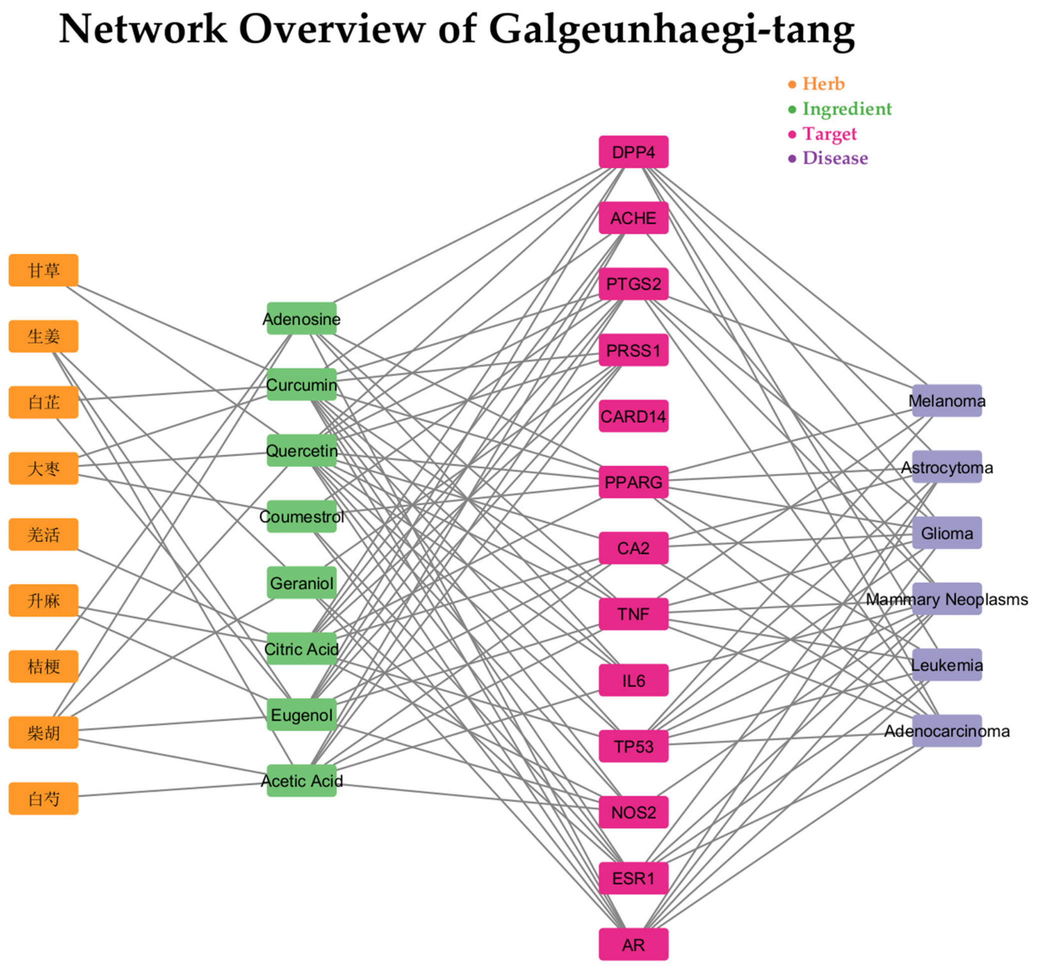


Below are the non-English herb names appearing in the figure. Each is provided with its standard Traditional Chinese Medicine name (Chinese characters) and Latin pharmaceutical name. 甘草 (Glycyrrhizae Radix et Rhizoma), 生姜 (Zingiberis Rhizoma Recens), 白芷 (Angelicae Dahuricae Radix), 大枣 (Jujubae Fructus), 羌活 (Notopterygii Rhizoma et Radix), 升麻 (Cimicifugae Rhizoma), 桔梗 (Platycodonis Radix), 柴胡 (Bupleuri Radix), 白芍 (Paeoniae Radix Alba)

**Supplementary Figure S4.** Network Overview of Daeshiho-tang.

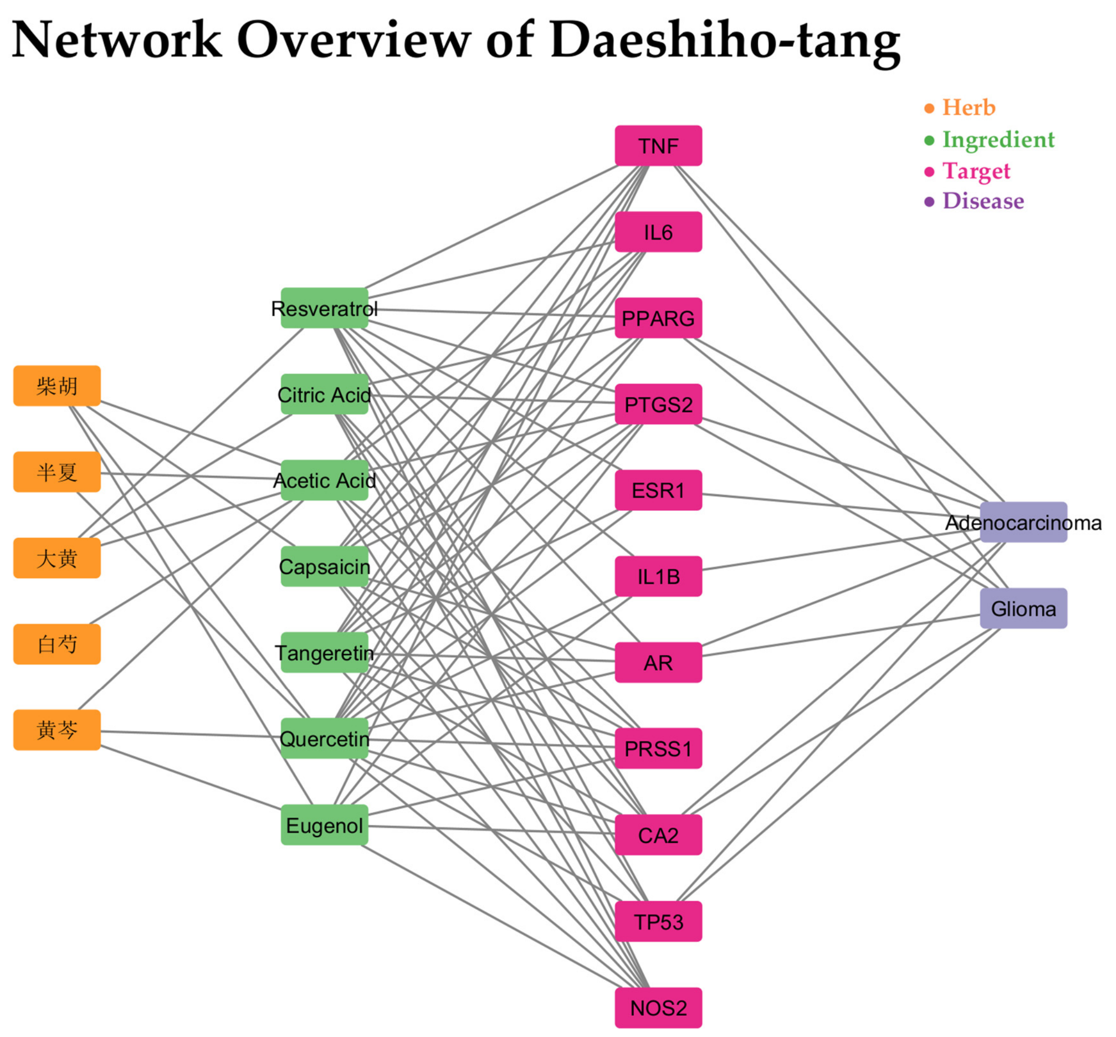


Below are the non-English herb names appearing in the figure. Each is provided with its standard Traditional Chinese Medicine name (Chinese characters) and Latin pharmaceutical name. 柴胡 (Bupleuri Radix), 半夏 (Pinelliae Rhizoma), 大黃 (Rhei Radix et Rhizoma), 白芍 (Paeoniae Radix Alba), 黃芩 (Scutellariae Radix)

**Supplementary Figure S5.** Network Overview of Banhabakchulcheonma-tang.

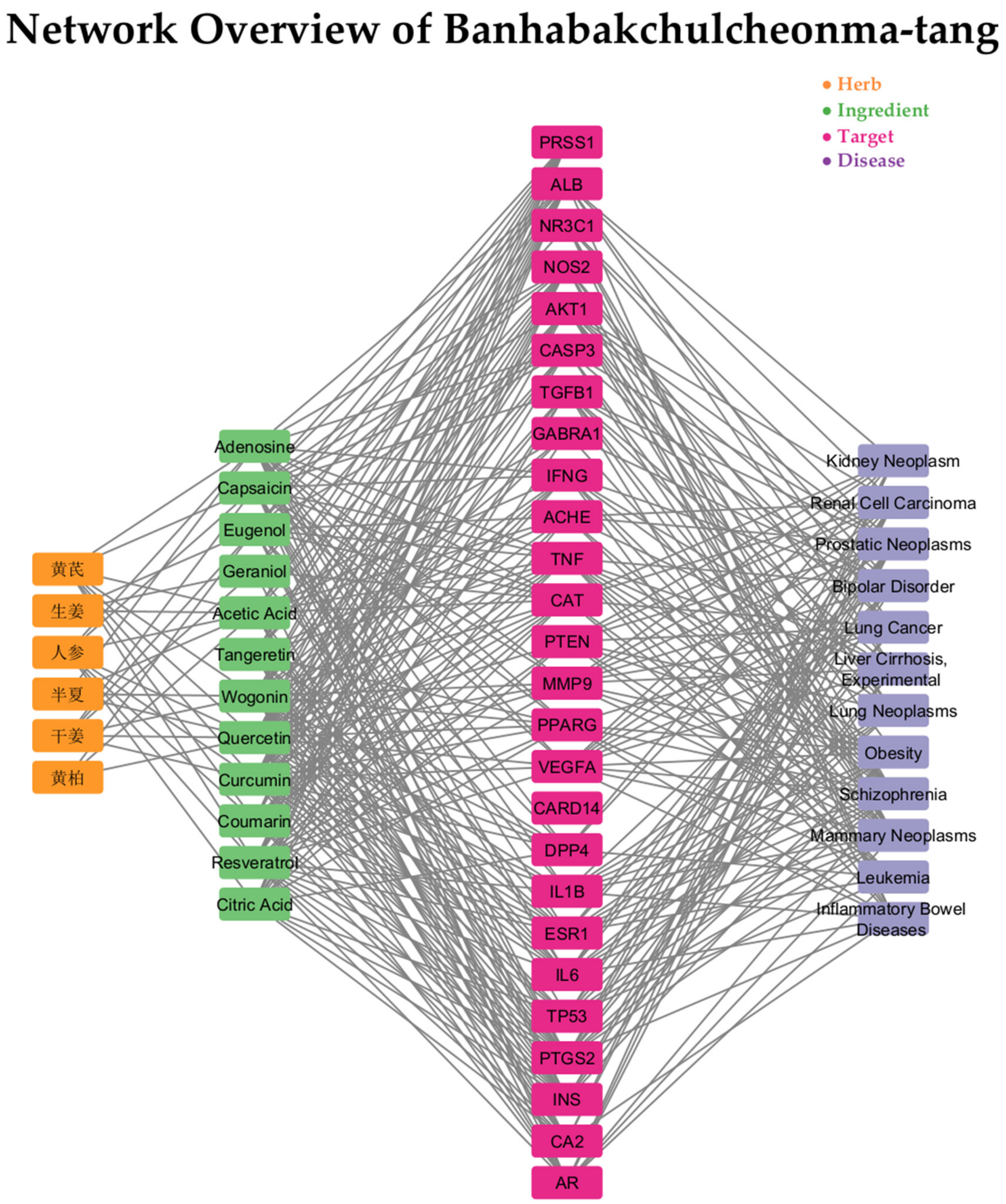


Below are the non-English herb names appearing in the figure. Each is provided with its standard Traditional Chinese Medicine name (Chinese characters) and Latin pharmaceutical name. 黃芪 (Astragali Radix), 生姜 (Zingiberis Rhizoma Recens), 人参 (Ginseng Radix Et Rhizoma), 半夏 (Pinelliae Rhizoma), 干姜(Zingiberis Rhizoma, 黄柏 (Phellodendri Chinensis Cortex)

**Supplementary Figure S6.** Network Overview of Banhasasim-tang.

# Network Overview of Banhasasim-tang

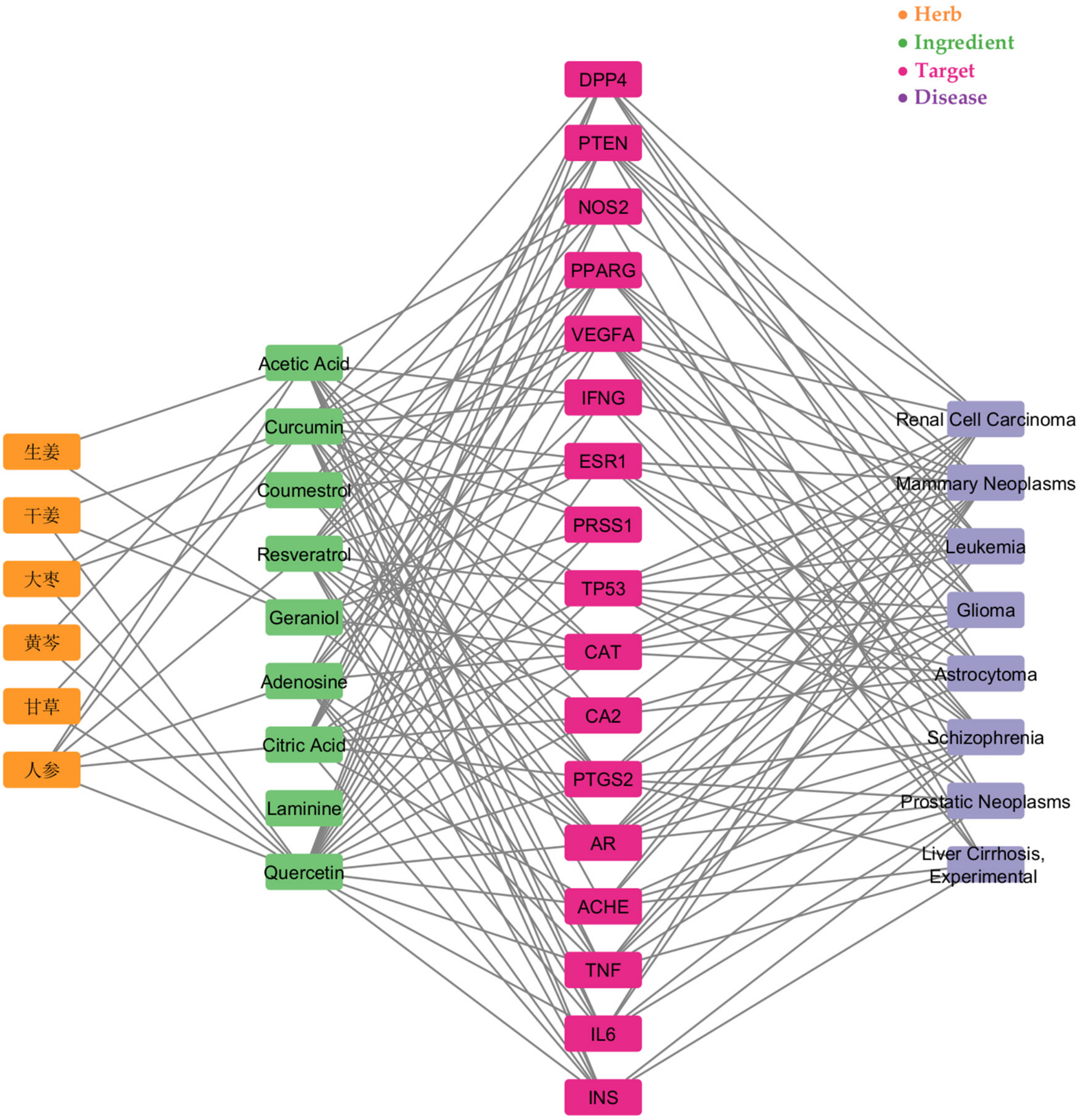


Below are the non-English herb names appearing in the figure. Each is provided with its standard Traditional Chinese Medicine name (Chinese characters) and Latin pharmaceutical name. 生姜 (Zingiberis Rhizoma Recens), 干姜 (Zingiberis Rhizoma), 大枣 (Jujubae Fructus), 黄芩 (Scutellariae Radix), 甘草 (Glycyrrhizae Radix et Rhizoma), 人参 (Ginseng Radix Et Rhizoma)

**Supplementary Figure S7.** Network Overview of Banhahubak-tang.

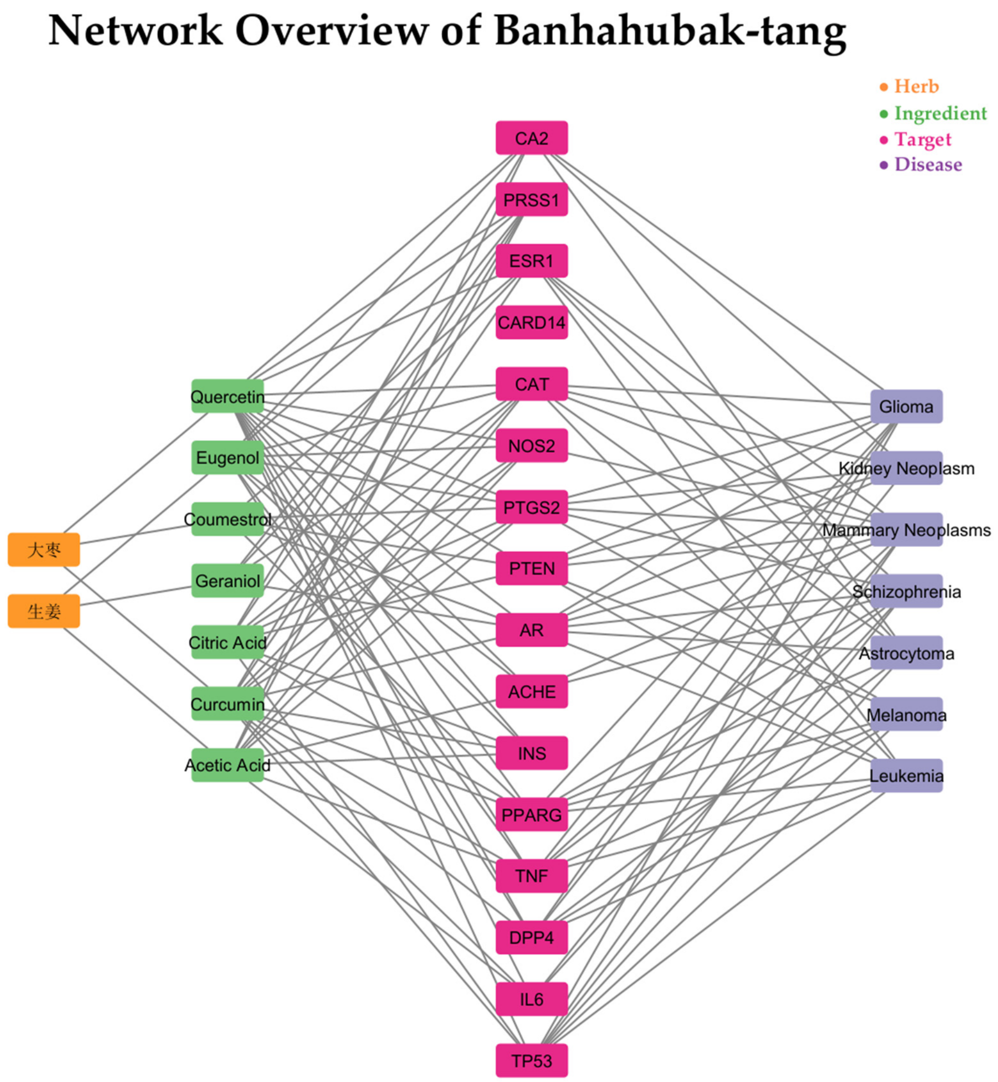


Below are the non-English herb names appearing in the figure. Each is provided with its standard Traditional Chinese Medicine name (Chinese characters) and Latin pharmaceutical name. 大枣 (Jujubae Fructus), 生姜 (Zingiberis Rhizoma Recens)

**Supplementary Figure S8.** Network Overview of Bojungikgi-tang.

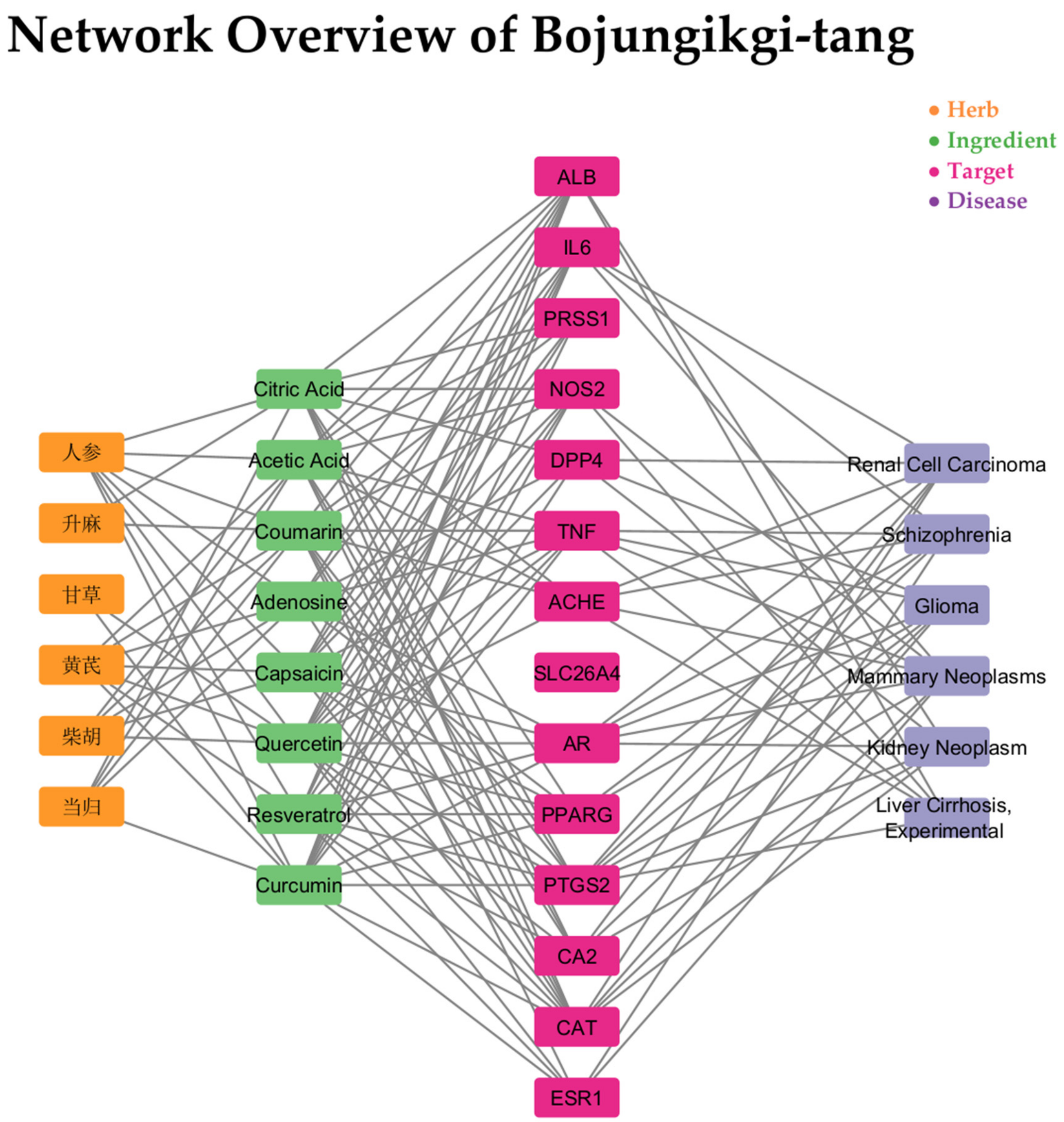


Below are the non-English herb names appearing in the figure. Each is provided with its standard Traditional Chinese Medicine name (Chinese characters) and Latin pharmaceutical name. 人参 (Ginseng Radix Et Rhizoma), 升麻 (Cimicifugae Rhizoma), 甘草 (Glycyrrhizae Radix et Rhizoma), 黃芪 (Astragali Radix), 柴胡 (Bupleuri Radix), 当归 (Angelicae Sinensis Radix)

**Supplementary Figure S9.** Network Overview of Saengmaek-san.

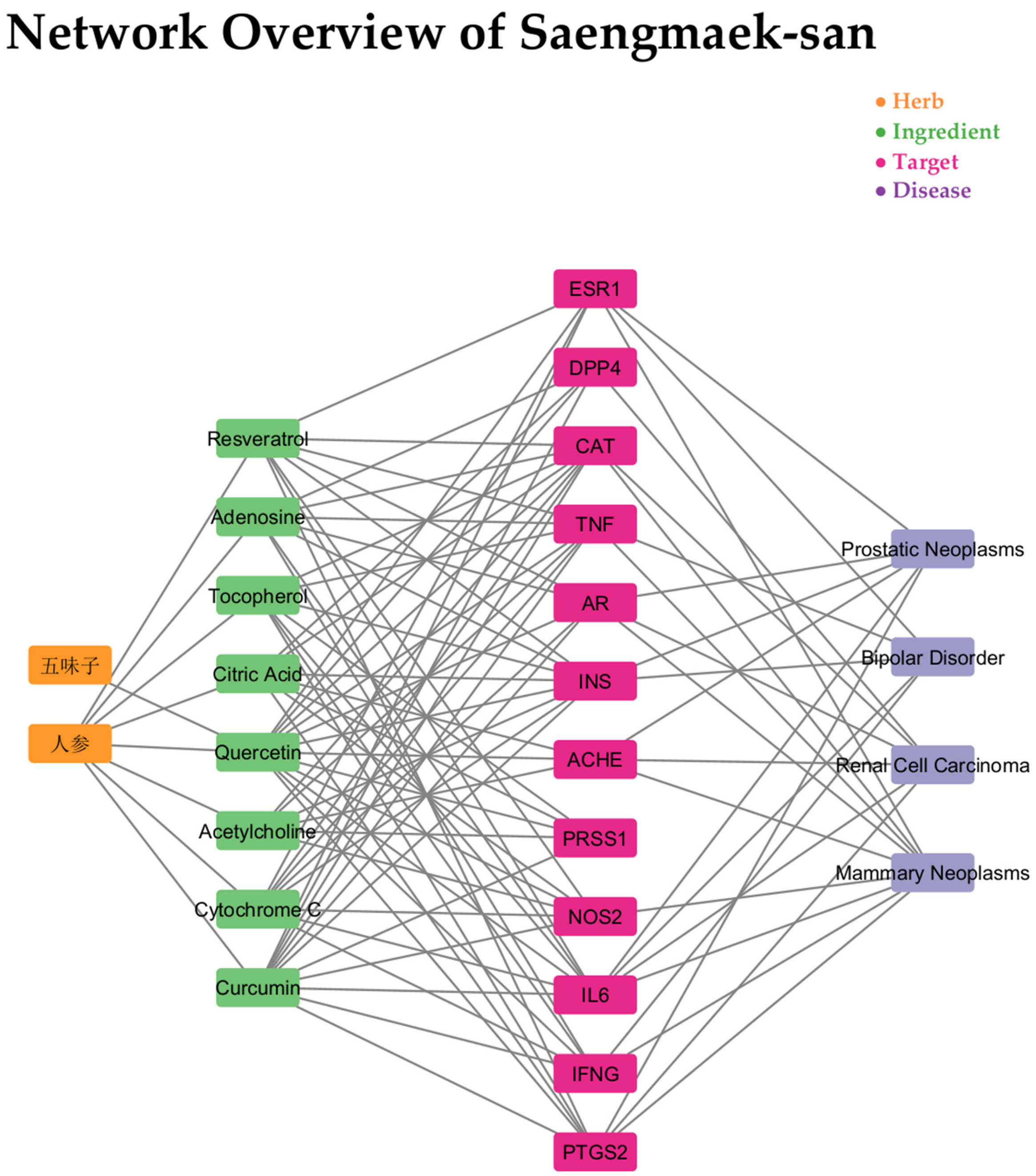


Below are the non-English herb names appearing in the figure. Each is provided with its standard Traditional Chinese Medicine name (Chinese characters) and Latin pharmaceutical name. 五味子 (Schisandrae Chinensis Fructus), 人参 (Ginseng Radix Et Rhizoma)

**Supplementary Figure S10.** Network Overview of Sosiho-tang.

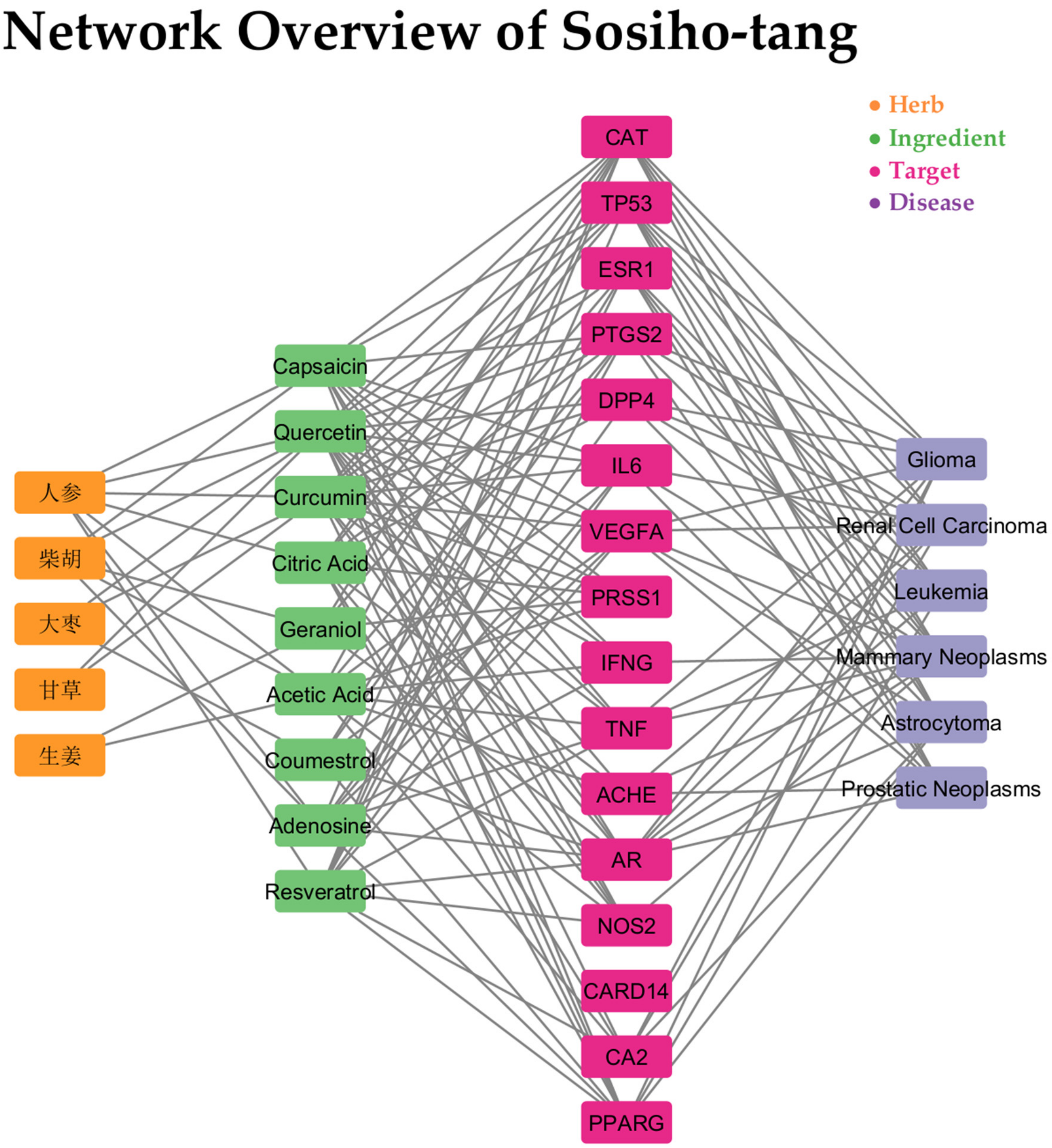


Below are the non-English herb names appearing in the figure. Each is provided with its standard Traditional Chinese Medicine name (Chinese characters) and Latin pharmaceutical name. 人参 (Ginseng Radix Et Rhizoma), 柴胡 (Bupleuri Radix), 大枣 (Jujubae Fructus), 甘草 (Glycyrrhizae Radix et Rhizoma), 生姜 (Zingiberis Rhizoma Recens)

**Supplementary Figure S11.** Network Overview of Socheongryong-tang.

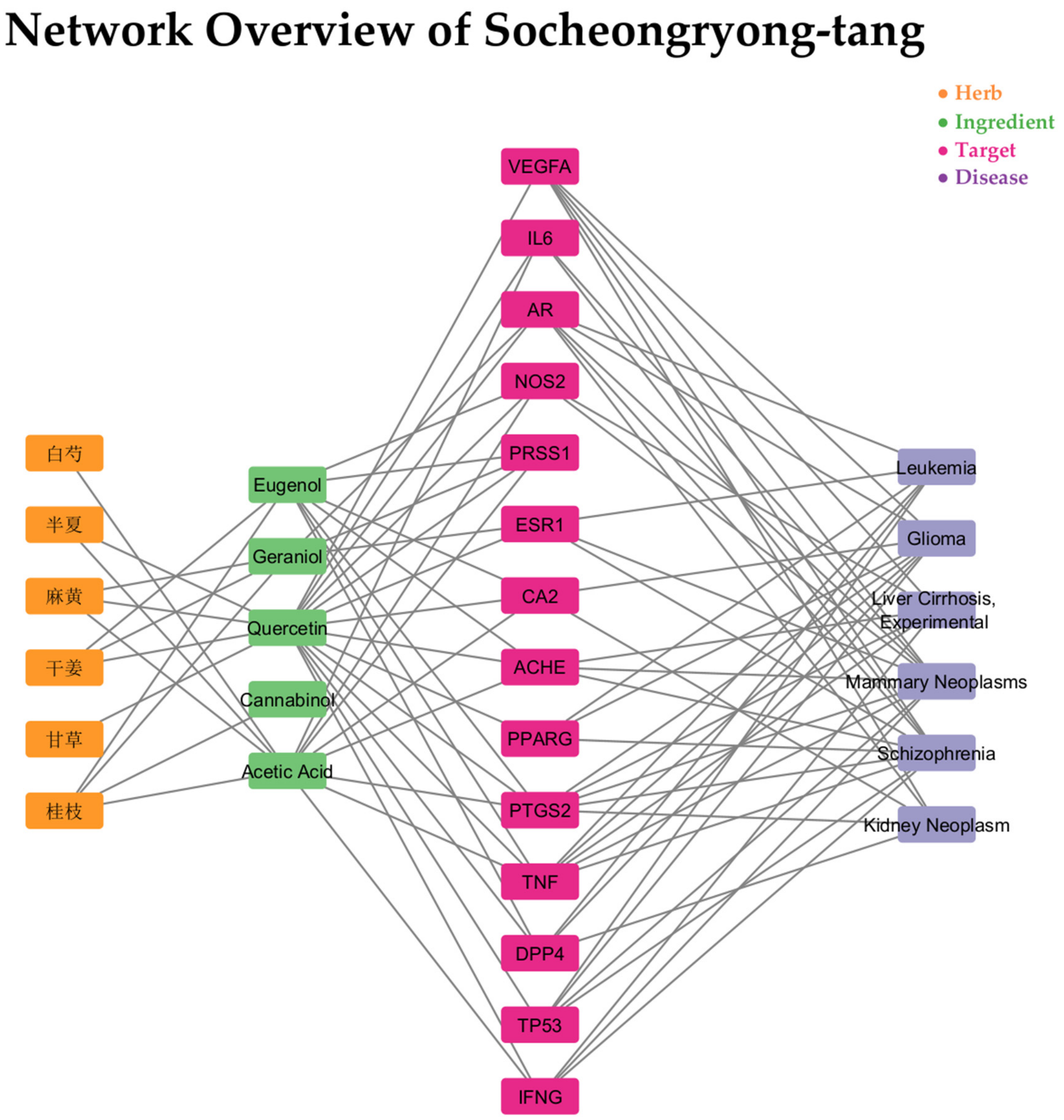


Below are the non-English herb names appearing in the figure. Each is provided with its standard Traditional Chinese Medicine name (Chinese characters) and Latin pharmaceutical name. 白芍 (Paeoniae Radix Alba), 半夏 (Pinelliae Rhizoma), 麻黄 (Ephedrae Herba), 干姜 (Zingiberis Rhizoma), 甘草 (Glycyrrhizae Radix et Rhizoma), 桂枝 (Cinnamomi Ramulus)

**Supplementary Figure S12.** Network Overview of Hyeonggaeyeongyo-tang.

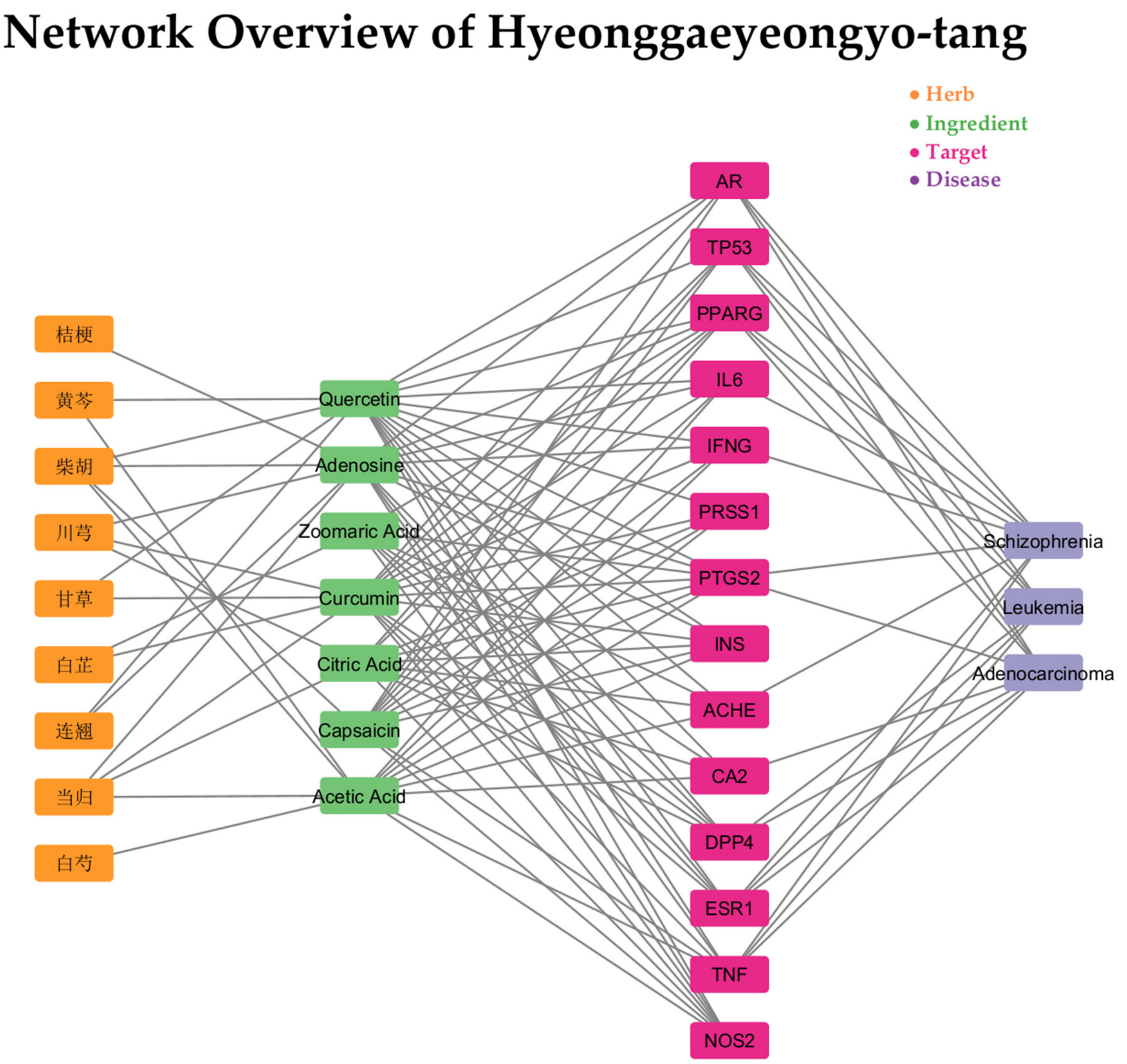


Below are the non-English herb names appearing in the figure. Each is provided with its standard Traditional Chinese Medicine name (Chinese characters) and Latin pharmaceutical name. 桔梗 (Platycodonis Radix), 黃芩 (Scutellariae Radix), 柴胡 (Bupleuri Radix), 川芎 (Chuanxiong Rhizoma), 甘草 (Glycyrrhizae Radix et Rhizoma), 白芷 (Angelicae Dahuricae Radix), 连翘 (Forsythiae Fructus), 当归 (Angelicae Sinensis Radix), 白芍 (Paeoniae Radix Alba)

**Supplementary Figure S13.** Network Overview of Hwanglyeonhaedok-tang.

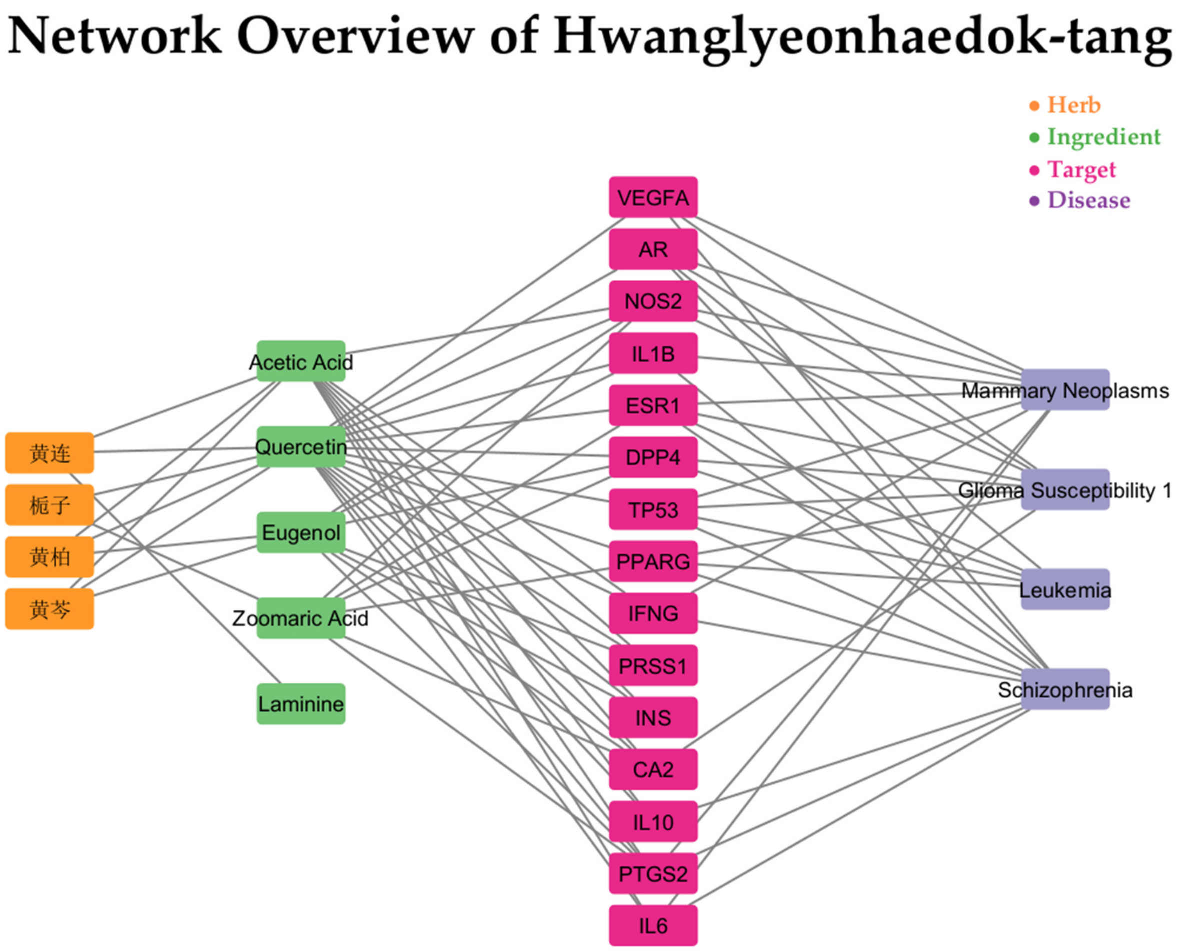


Below are the non-English herb names appearing in the figure. Each is provided with its standard Traditional Chinese Medicine name (Chinese characters) and Latin pharmaceutical name. 黄连 (Coptidis Rhizoma), 黄芩 (Scutellariae Radix), 黄柏 (Phellodendri Chinensis Cortex), and 栀子 (Gardeniae Fructus).
