# Supplementary material for: Network Analysis of Predicted Therapeutic Symptoms in National Health Insurance Herbal Prescriptions
Source: Life (Basel). 2025 Nov 18;15(11):1769. doi: 10.3390/life15111769 (PMC12654001; doi:10.3390/life15111769)
Supplement: Supplementary file 1 [file life-15-01769-s001.zip › life-3962368-supplementary/Supplementary Table S1.pdf]

**Supplementary Table S1.** Main indication of the 56 prescriptions

| Prescription | Main indication |
|---|---|
| 1. Gamisoyo-san | Various physical and mental symptoms before and during menstruation, dysmenorrhea, menorrhagia, polymenorrhea, oligomenorrhea, irregular menstruation, postpartum depressive mood and depression, perimenopausal and menopausal physical and mental symptoms, pruritus, stomatitis, dry mouth, sleep disorders, dizziness, fatigue, lethargy, anorexia, alternating chills and fever, cold extremities, unspecified fever or heat sensation, generalized body aches and stiffness, shoulder stiffness, headache, night sweats, ear pain, chest/breast/abdominal distension, dysuria, blood-tinged sputum, psychogenic cough, neurosis |
| 2. Galgeun-tang | Common cold, myalgia, stiff neck and back pain, headache and facial pain, thirst, diarrhea, skin rash, rhinitis, sinusitis, acute bronchitis, acute laryngitis, scarlet fever, colitis |
| 3. Galgeunhaegi-tang | Internal heat syndrome (Yangming meridian disorder) causing eye pain and nasal dryness, sleep disorders, common cold, influenza, allergic rhinitis, atrophic rhinitis, acute sinusitis, allergic contact dermatitis, erythema multiforme |
| 4. Gumiganghwal-tang | Generalized body aches, common cold, influenza, contusion, various arthritis and arthralgia, beriberi |
| 5. Gungso-san | Pregnancy or postpartum common cold, myalgia, cough, asthma, influenza, bronchitis |
| 6. Gungha-tang | Digestive disorders with pathological fluid retention (phlegm-dampness): dyspepsia, ascites, abdominal pain, hypochondriac pain, chest pain, arthritis, lumbosacral pain, anxiety, pleurisy, obstructive lung disease |
| 7. Naeso-san | Indigestion, nausea, vomiting, abdominal bloating, epigastric discomfort, constipation, diarrhea, abdominal pain, anorexia, low back pain, esophagitis, esophageal ulcer, acute/chronic gastroduodenitis, peptic ulcer, colitis, irritable bowel syndrome, intussusception, chronic cholecystitis, alcoholic gastritis, alcoholic liver disease, throat nodules, thyroid nodules, depressive mood, psychosomatic disorder, globus hystericus |

| | |
|---|---|
| 8. Dang-gwi-yeon-gyo-eum | Toothache, halitosis, dry mouth/lips, stomatitis, gingivitis, periodontitis, oral thrush, angular cheilitis, herpes labialis |
| 9. Dang-gwi-yuk-hwang-tang | Night sweats, cold sweats, postpartum excessive sweating, postpartum wind-induced sweating, fluid loss with heat sensation, constipation, dark urine, red tongue, rapid pulse, facial flushing, chest/palm/sole heat, perimenopausal symptoms, fatigue, headache, vertigo, migraine, psychosomatic disorder, autonomic dysfunction, anemia, chronic wasting diseases |
| 10. Daeshiho-tang | Delirium, hiccups, obesity, constipation, thirst, chest pain, palpitations, fever of unknown origin, periodic fever, hematuria, halitosis, glossalgia, common cold, myalgia, abdominal distension, nausea, vomiting, epidemic keratoconjunctivitis, acute conjunctivitis, stomatitis, periodontitis, glossitis, acute tonsillitis, acute laryngitis, acute tracheitis, acute bronchitis, bronchiolitis, acute pharyngolaryngitis, otitis media, sinusitis, influenza, pneumonia, asthma, pulmonary gangrene, chronic obstructive pulmonary disease, hepatitis, cholecystitis, cholangitis, peptic ulcer, acute appendicitis, acute pancreatitis, peritonitis, irritable bowel syndrome, gastrointestinal malignancies, chronic ischemic heart disease, schizophrenia, dissociative disorder |
| 11. Daecheongryoung-tang | Fever, chills, body aches, chest tightness without sweating, common cold, myalgia, wheezing, acute pharyngitis, acute tonsillitis, acute laryngitis, acute tracheitis, acute bronchitis, bronchiolitis, acute laryngotracheitis, croup, influenza, pneumonia, chronic obstructive pulmonary disease, pulmonary edema, acute sinusitis, acute conjunctivitis, erysipelas |
| 12. Daehwa-jung-eum | Indigestion, anorexia, abdominal distension, abdominal mass sensation, constipation, diarrhea, alcohol use disorder, gastroduodenitis, esophagitis, colitis, malabsorption syndrome, peptic ulcer, pyloric spasm, acute gastric dilatation, post-surgical digestive complications |
| 13. Daehwangmokdanpi-tang | Constipation, abdominal pain, acute appendicitis, intestinal inflammation, peptic ulcer (in robust patients), hemorrhoids, pelvic inflammatory disease, dysmenorrhea |
| 14. Doinseunggitang | Constipation, lower abdominal tension, black stools, dysuria, hematuria, epistaxis, headache, pre-stroke symptoms, fever of unknown origin, high fever with unconsciousness, delirium, |

| | |
|---|---|
| | dysmenorrhea, hypomenorrhea, pelvic inflammatory disease, chronic nephritis, cystitis, alcohol use disorder, dissociative disorder, schizophrenia |
| 15. Banhabaekchulcheonma-tang | Dyspepsia, tinnitus with digestive weakness, dizziness, headache, migraine, nausea, chest tightness, tachypnea, vomiting, periorbital hyperpigmentation, chronic gastritis |
| 16. Banhasasim-tang | Epigastric fullness without pain, fever with vomiting (infections), stomatitis, hangover, dyspepsia, anorexia, nausea, vomiting, acute gastric dilatation, gastroptosis, heartburn, belching, peptic ulcer, esophagitis, gastroduodenitis, neurogenic gastritis, colitis, functional dyspepsia, pyloric spasm, malabsorption syndrome, diarrhea, chronic ischemic heart disease, neurosis |
| 17. Banhahubak-tang | Dyspepsia, anorexia, nausea, vomiting, abdominal distension, gastritis, gastric ulcer, neurogenic cough, globus hystericus, hoarseness, benign pharyngeal tumors, fatigue, dizziness, palpitations, depressive mood, anxiety neurosis, morning sickness, perimenopausal symptoms |
| 18. Backchul-tang | Productive cough, chronic bronchitis, dyspnea, post-illness fatigue, chronic diarrhea, anorexia, heavy body sensation, unspecified pain, prolonged vomiting/diarrhea with thirst and cramps, polydipsia, leg cramps, hyperhidrosis, pregnancy/postpartum diarrhea or dysentery |
| 19. Bojungikgi-tang | Dyspepsia, abdominal pain, functional diarrhea, gastroduodenitis, peptic ulcer, gastroptosis, chronic intestinal ischemia, post-illness fatigue, chronic fatigue syndrome, hyperhidrosis, cold sweats, heat sensation with fatigue, dysuria, cystitis, chronic nephritis, chronic cough, chronic laryngotracheitis, hiccups, limb paralysis, CNS inflammatory sequelae, neuralgia, hypothalamic dysfunction, paraplegia, menorrhagia, polymenorrhea, cervical cancer sequelae, urinary incontinence, genital prolapse, leukorrhea, vulvovaginal disorders, gestational diabetes, miscarriage, early pregnancy bleeding, obstetric trauma, perineal laceration, non-inflammatory vulvar disorders, pregnancy-related digestive disorders, sexual dysfunction, galactorrhea, hearing loss, tinnitus, chronic tympanitis, skin abscess, anxiety, tension |

| | |
|---|---|
| 20. Boheotang | Postpartum disorders, postpartum anemia, persistent somatic pain, pregnancy/postpartum infections, lactation-related breast disorders |
| 21. Bokryeongbosim-tang | Chest/palm/sole sweating, fatigue, flushing, stress-induced hematemesis or sweating, epistaxis, polydipsia, nausea, vomiting, gastrointestinal bleeding, anemia, hypertension, hypotension, tuberculosis, perimenopausal symptoms, mood disorders, anxiety, cognitive dysfunction, neurodegenerative disorders |
| 22. Bulhwan-geumjeonggi-san | Nausea, vomiting, diarrhea, food/water poisoning, cholera-like symptoms, alternating chills and fever, cough, esophagitis, gastroduodenitis, colitis, functional bowel disorder, dysentery, food poisoning, common cold, influenza, laryngotracheitis, bronchitis, bronchiectasis, beriberi |
| 23. Samso-eum | Cough, rhinorrhea, sneezing, nasal congestion, post-infectious abnormal movements, periodic fever, psychogenic chest tightness, common cold, influenza, acute laryngotracheitis, pregnancy/postpartum skin disorders, cholecystitis, respiratory neoplasms (symptomatic management) |
| 24. Samchulgeonbi-tang | Dyspepsia (weak digestive system), anorexia, neurogenic anorexia, abdominal distension, peptic ulcer, esophagitis, gastroduodenitis, colitis, malabsorption syndrome, functional diarrhea, irritable bowel syndrome with diarrhea, achalasia, esophageal dysmotility, esophageal neoplasms, pyloric spasm, chronic viral hepatitis |
| 25. Sam-ho-jak-yak-tang | Thirst, anxiety, constipation, dysuria, urinary retention, recurrent hematuria, acute/chronic nephritis, nephrotic syndrome, glomerular disorders, urethral syndrome, acute prostatitis, urethral stricture, acute cystitis, interstitial cystitis, post-infectious residual fever |
| 26. Samhwangsa-sim-tang | Facial flushing (robust patients), anxiety, constipation, hypertension-related shoulder stiffness, tinnitus, heavy-headedness, insomnia, epigastric tightness, cardiac dysfunction, fever of unknown origin, epistaxis, hematemesis, recurrent stomatitis, tongue papillary hypertrophy, angina, pruritus |

| | |
|---|---|
| 27. Saengmaek-san | Summer heat-related fatigue, heatstroke, gastrointestinal upset, fatigue, dyspnea, dry mouth, hyperhidrosis, acute pharyngitis, voice disorders, laryngeal conditions |
| 28. Sosiho-tang | Bitter taste, chest/hypochondriac fullness, anorexia, nausea, cold sweats, night sweats, alternating chills and fever, vertigo, hearing loss, prolonged post-cold fever, chronic tonsillitis, influenza, acute/chronic laryngotracheitis, psychogenic cough, asthma, bronchiectasis, gastritis, acute cholecystitis, acute pancreatitis, esophageal dysmotility, pregnancy-related liver dysfunction, viral hepatitis, pleurisy, diaphragmatic disorders, amenorrhea/menopause-related fever, menstrual fever, postpartum fever, pelvic inflammatory disease, acute cervicitis, oophoritis, acute female pelvic inflammation, intercostal neuralgia, psychosomatic disorder, acute nephritis, recurrent hematuria, chronic nephritis, interstitial nephritis, cystitis, urethritis, prostatitis, male genital inflammation, hematuria, nephrotic syndrome, glomerulopathy, delirium, depressive episode |
| 29. Socheongryong-tang | Common cold, rhinorrhea, sneezing, cough, pleuritic pain, epigastric pain, hiccups, nausea, thirst with diarrhea, arthralgia, rhinitis, acute/chronic pharyngitis, influenza, asthma, pneumonia, pleurisy, pulmonary edema, interstitial lung disease, pleural effusion, respiratory neoplasms (symptomatic management), acute conjunctivitis |
| 30. Seungyang-bo-wi-tang | Diarrhea, mucous/bloody stools, gastrointestinal bleeding, tenesmus, heavy body sensation, bitter/dry mouth, anorexia, fatigue, constipation, frequent urination, cold intolerance, malabsorption, dysentery, gastroenteritis, colitis, gastrointestinal neoplasms (symptomatic management) |
| 31. Sikyungbanha-tang | Fever, cough, chest congestion, hypochondriac pain, productive cough, acute tracheitis, laryngotracheitis, influenza, pneumonia, digestive neoplasms (symptomatic management) |
| 32. Sihogyeji-tang | Common cold, alternating chills and fever, abdominal pain, hypochondriac/chest pain, headache, neck stiffness, hyperhidrosis, polydipsia, intercostal neuralgia, peptic ulcer, acute/chronic hepatitis, cholecystitis, stress-related pain, psychosomatic disorder |

| | |
|---|---|
| 33. Shihosogan-tang | Indigestion or trauma-induced hypochondriac pain, alternating fever/chills, pleurisy, intercostal neuralgia, pancreatic disorders, psychosomatic disorder, depressive episode |
| 34. Si-ho-cheong-gan-Tang | Irritability, anger, ear/neck/breast/hypochondriac/chest pain, alternating chills/fever, acute hepatitis, pleurisy, pediatric chronic tonsillitis with irritability, eczema, atopic dermatitis, hypersensitivity, psychosomatic disorder |
| 35. Antae-eum | Pregnancy-related abdominal pain, vaginal bleeding, miscarriage, recurrent miscarriage, preterm labor |
| 36. Yeongyopaedok-san | Acute upper respiratory infections (e.g., common cold), mumps, abscess-related chills/fever/headache/edema |
| 37. Oryeong-san | Dysuria, hematuria, urethritis, cystitis, renal/ureteral calculi with dysuria/pyuria/hematuria/lower abdominal pain, acute/chronic nephritis, nephrotic syndrome |
| 38. Ojeok-san | Low back pain, sciatica, arthralgia, contusion, common cold, headache, facial palsy, neuralgia, dysmenorrhea, cold sensation, leukorrhea, nausea, vomiting, dyspepsia, eye strain, postpartum disorders, lumbar sprain, hip osteoarthritis, ankle sprain, neck sprain, beriberi, muscle strain, synovitis, tenosynovitis, Paget's disease (symptomatic management), cerebrovascular sequelae, toxic encephalopathy, neuropathy, perimenopausal symptoms, premenstrual syndrome, uterine fibroids (symptomatic management), gynecologic malignancies (symptomatic management), female genital inflammatory disorders (adjuvant), irritable bowel syndrome, conjunctivitis, depressive episode, recurrent depressive disorder, dysthymia, acute stress reaction, adjustment disorder, hypochondriasis, neurasthenia, psychosomatic disorder |
| 39. Yijung-tang | Abdominal pain/diarrhea without thirst, nausea, sensory disturbances, cold intolerance, muscle tension, low back pain, lumbosacral pain, chronic cough (weak patients), gastroduodenitis, peptic ulcer, colitis, functional diarrhea, irritable bowel syndrome, pregnancy/postpartum respiratory disorders, hip osteoarthritis |
| 40. Yijin-tang | Poor qi-blood circulation-related symptoms: dyspepsia, nausea, mild morning sickness, dizziness, palpitations, temperature intolerance, migratory pain, chronic gastritis, |

| | |
|---|---|
| | pregnancy/postpartum digestive disorders, neck sprain, CNS inflammatory sequelae, pleurisy |
| 41. Yikwiseungyangtang | Fatigue due to blood loss, post-hemorrhage recovery, anxiety, menorrhagia, polymenorrhea, irregular menstruation, dysfunctional uterine bleeding, endometrial hyperplasia, miscarriage/ectopic pregnancy complications |
| 42. Insampaedok-san | High fever with chills, headache, eye pain, generalized pain, cough, nasal congestion, myalgia, overexertion-related pain, common cold, acute tracheitis, acute bronchitis, chronic bronchitis, pneumonia |
| 43. Injinho-tang | Jaundice, thirst, constipation, urticaria, hepatitis, stomatitis |
| 44. Ja-eumganghwa-tang | Cough, hemoptysis, dysuria, fever with fluid loss, flushing, night sweats, weight loss, anorexia, chronic laryngotracheitis, chronic bronchitis, bronchiectasis, depressive episode, recurrent depressive disorder, persistent mood disorder |
| 45. Jowiseung-gi-tang | Constipation with chest/abdominal distension, delirium with diarrhea, periodic fever, mucopurulent stools, thirst, acute appendicitis, erythema multiforme, delirium, schizophrenia, acute transient psychotic disorder |
| 46. Cheongsanggyeon-tong-tang | Headache, facial pain, vertigo, anxiety disorders |
| 47. Cheongseoikgitang | Heat intolerance, hyperhidrosis, summer heat-induced fatigue, thirst, anorexia, heat sensation, diarrhea, dysentery, summer common cold, influenza |
| 48. Cheongwee-San | Gastric heat-related recurrent aphthous ulcers, stomatitis, toothache, gingivitis, periodontitis |
| 49. Palmul-tang | Fatigue in debilitated patients, anorexia, tinnitus, post-illness recovery, anemia, hypotension, leg cramps, numbness, neuralgia, neuritis, hematuria, hypomenorrhea, oligomenorrhea, menorrhagia, polymenorrhea, irregular menstruation, dysmenorrhea, delayed puberty, amenorrhea, transient cerebral ischemia, cerebrovascular disease, acute ischemic heart disease, cardiac arrhythmia, malignant neoplasms (symptomatic management) |
| 50. Pyeongwi-san | Acute/chronic indigestion, dyspepsia, acute gastric dilatation, abdominal distension, heartburn, abdominal pain, nausea, vomiting, diarrhea, pregnancy/postpartum |

| | |
|---|---|
| | nausea/vomiting/indigestion/diarrhea, fatigue, cough, low back pain, lumbosacral pain, GERD, esophagitis, gastroduodenitis, peptic ulcer, colitis, irritable bowel syndrome, malabsorption syndrome, achalasia, esophageal dysmotility, summer common cold, gastrointestinal neoplasms (symptomatic management) |
| 51. Haengso-tang | Common cold, cough, sputum, chronic laryngotracheitis |
| 52. Hyangsapyeongwi-san | Dyspepsia, diarrhea, nausea, vomiting, heartburn, abdominal pain, acute/chronic indigestion, esophagitis, gastroduodenitis, summer common cold, depressive episode |
| 53. Hwangkeumjagyag-tang | Bloody stools, fever with abdominal pain, dysentery, infectious colitis with mucoid stools, gastroduodenitis, appendicitis |
| 54. Hwanglyeonhaedok-tang | High fever with inflammation (robust patients), infection-related fever, seizures, confusion, insomnia, cough, epistaxis, stomatitis, glossitis, tinnitus, dizziness, palpitations, alcoholic gastritis, alcoholic liver disease, irritant contact dermatitis, erysipelas, acute conjunctivitis, schizophrenia, bipolar disorder, dissociative disorder, neurosis |
| 55. Hyeonggaeyeongyo-tang | Nasal congestion, rhinorrhea, nasal pruritus, cough, asthma, ear pain, otitis externa, common cold, influenza, rhinitis, allergic rhinitis, sinusitis, pharyngitis, tonsillitis, bronchiolitis, bronchitis, tracheitis, laryngotracheitis, pertussis, otitis media, tympanitis |
| 56. Hoichunyanggyuksan | Constipation, palm/sole heat, epistaxis, headache, fissured tongue, abdominal pain, facial flushing, acne, fever of unknown origin, thirst, stomatitis, glossitis, oral thrush, gingivitis, periodontitis, pharyngitis, peptic ulcer, gastroduodenitis, perimenopausal symptoms |

NHP, National Health Insurance herbal prescriptions
